# Molecular organization of the New World arenavirus spike glycoprotein complex

**DOI:** 10.1038/s41564-025-02085-6

**Published:** 2025-08-08

**Authors:** Colin J. Mann, Pan Yang, Daniel Olal, Xiaoyi Fan, Katherine Nabel Smith, Lars E. Clark, Florian Krammer, Yuemin Bian, Jonathan Abraham

**Affiliations:** 1 Department of Microbiology, Blavatnik Institute, Harvard Medical School, Boston, MA USA; 2 Department of Microbiology, Icahn School of Medicine at Mount Sinai, New York, NY USA; 3 Department of Pathology, Molecular and Cell Based Medicine, Icahn School of Medicine at Mount Sinai, New York, NY USA; 4 Center for Vaccine Research and Pandemic Preparedness (C-VARPP), Icahn School of Medicine at Mount Sinai, New York, NY USA; 5 Ignaz Semmelweis Institute, Interuniversity Institute for Infection Research, Medical University of Vienna, Vienna, Austria; 6 School of Medicine, Shanghai University, Shanghai, China; 7 Department of Medicine, Division of Infectious Diseases, Brigham and Women’s Hospital, Boston, MA USA; 8 Center for Integrated Solutions for Infectious Diseases, Broad Institute of Harvard and MIT, Cambridge, MA USA; 9 Howard Hughes Medical Institute, Boston, MA USA

**Keywords:** Arenaviruses, Viral membrane fusion, Cryoelectron microscopy

## Abstract

Of the multiple arenaviruses that cause haemorrhagic fevers in the Americas, all lack reliable therapeutic options, and only one has a vaccine. The arenavirus glycoprotein complex (GPC) binds cellular receptors and mediates pH-dependent fusion of viral and host cell membranes during entry. GPC comprises GP1, GP2 and stable signal peptide (SSP) subunits. SSP remains associated with the mature glycoprotein complex and regulates pH-dependent membrane fusion through an unclear mechanism. We report cryo-EM structures of Junin virus and Machupo virus GPC stabilized in the prefusion conformation using an amino acid substitution in the transmembrane region of SSP at 3.0 Å and 2.9 Å resolution, respectively. Mutational analyses, cell–cell fusion assays and molecular dynamics simulations reveal how contacts in the membrane-proximal and transmembrane regions of GPC regulate pH-dependent membrane fusion. The structures may aid in the design of therapeutic antibody cocktails, small-molecule inhibitors and vaccines against arenaviruses.

## Abstract (web summary)

Cryo-EM structures of the full-length Junin virus and Machupo virus spike glycoprotein complexes stabilized in the prefusion conformation. Analyses reveal features that regulate glycoprotein pH-dependent membrane fusion activity.

## Main

Arenaviruses can cause haemorrhagic fevers with high case fatality rates when they spill over from rodent reservoirs to humans. They fall into two groups, Old World and New World, based on their phylogeny and geographic distribution^1^. Arenaviruses that cause disease in South America include Junin virus (JUNV), which causes Argentine haemorrhagic fever (AHF), and Machupo (MACV), Guanarito, Chapare, Sabiá and Sabiá virus-like viruses^2,3^. All New World haemorrhagic fever arenaviruses, except JUNV, lack effective vaccines or medical countermeasures.

The arenavirus glycoprotein complex (GPC) contains three subunits: GP1, GP2 and the stable signal peptide (SSP)^4^. Cleavage by signal peptidase generates SSP, and cleavage by cellular subtilisin kexin isozyme-1/site 1 protease (SKI-1/S1P) generates GP1 and GP2 (refs. ^5–8^). GP1, GP2 and SSP form trimers of heterotrimers on virions^4^. The GP1 subunits of New World arenaviruses bind transferrin receptor 1 (TfR1)^9–11^. GP2 contains a transmembrane (TM) anchor and mediates fusion of viral and host cell membranes during entry. SSP is an unusually long signal peptide (58 amino acids) that is myristoylated and regulates pH-dependent membrane fusion through an unclear mechanism^4,12–14^.

JUNV has a live attenuated vaccine (Candid#1) that is used in endemic regions^15^. While the major determinant of Candid#1 attenuation is a substitution in the JUNV GP2 TM region (F427I_GP2_), the mechanism of attenuation resulting from this substitution is unknown^16,17^. MACV does not have a vaccine, and although recombinant MACV containing the substitution that is analogous to JUNV F427I_GP2_ (MACV F438I_GP2_) is attenuated, the resulting virus is genetically unstable and can revert to wild type (WT) to regain pathogenicity^18^.

Here we report cryo-electron microscopy (cryo-EM) structures of JUNV and MACV GPC stabilized in the prefusion conformation through an amino acid substitution in SSP. Cell–cell fusion assays and molecular dynamics (MD) simulations clarify the mechanisms through which SSP substitutions regulate pH-dependent membrane fusion with implications for vaccine design and antiviral development.

## Results

### Cryo-EM structure of JUNV GPC

**Fig. 1:**
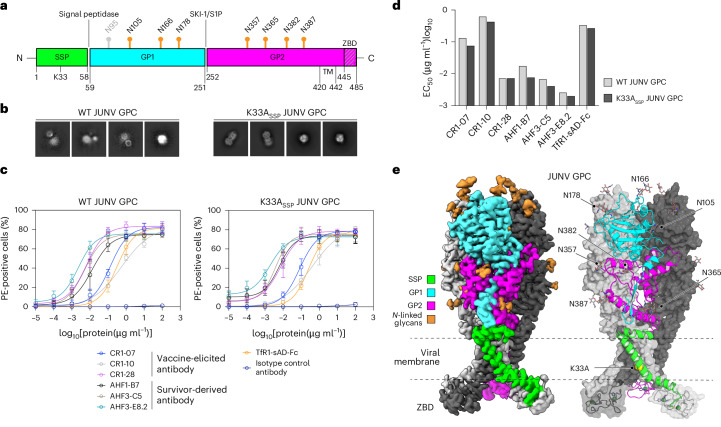
A stable signal peptide substitution stabilizes JUNV GPC in the prefusion conformation. **a**, Domain organization of JUNV GPC. Sites of *N*-linked glycosylation observed in cryo-EM maps are indicated. A putative glycan at GP1 N95, for which no density was observed, is shown in grey. Signal peptidase and SKI-1/S1P protease processing sites are shown. TM, transmembrane segment. ZBD, zinc-binding domain. **b**, 2D class averages of WT JUNV or K33A_SSP_ JUNV GPC. Each experiment was performed twice. Representative images are shown. See Extended Data Fig. 1a–c for additional information. **c**, Cell surface immunostaining of HEK 293T cells transiently transfected with WT or K33A_SSP_ JUNV GPC. Staining was performed using the indicated monoclonal antibodies or TfR1-sAD-Fc. Data are mean ± s.d. of 3 experiments, each performed in technical triplicate (*n* = 3 independent experiments). **d**, Half maximal effective concentration (EC_50_) values measured for immunostaining experiments with WT or K33A_SSP_ JUNV GPC from the experiment shown in **c**. **e**, Cryo-EM map (left) and model (right) of K33A_SSP_ JUNV GPC. For the model, one protomer is shown as a ribbon diagram and the others are shown as surfaces. The ZBDs found at the GP2 C termini are shown as ribbon diagrams with semi-transparent surfaces. Zinc ions are shown as green spheres. The positions of *N*-linked glycans, the K33A_SSP_ substitution and the viral membrane are indicated. Source data

**Extended Data Fig. 1:**
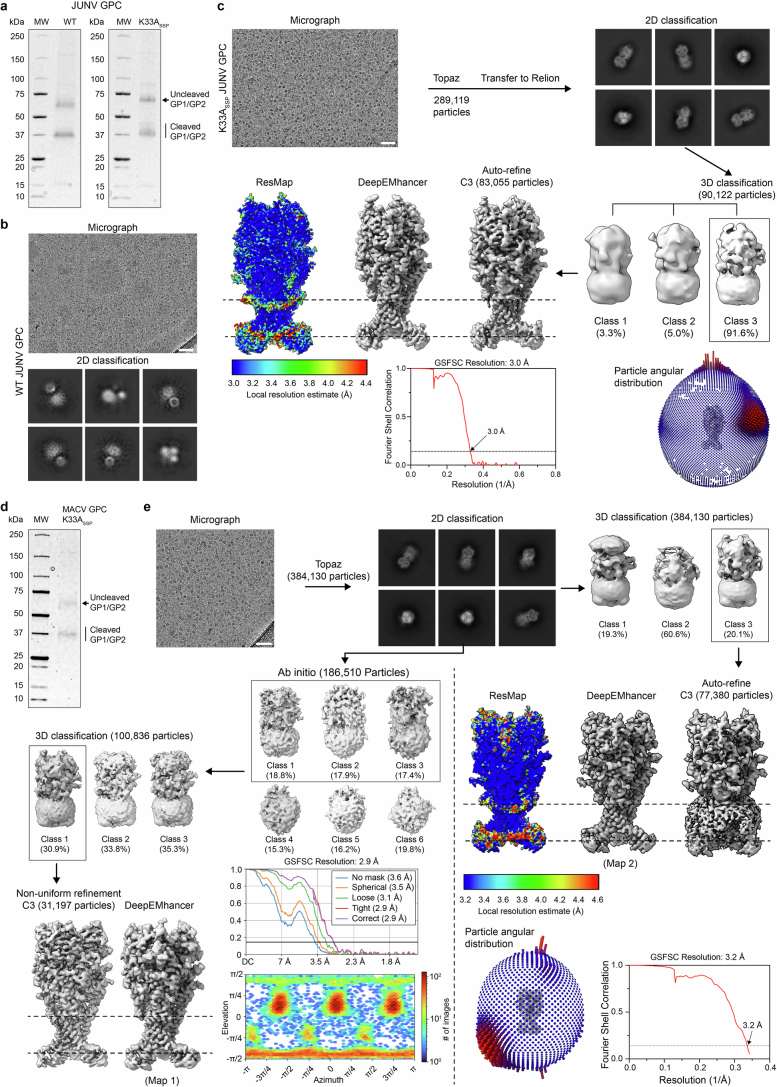
Cryo-EM reconstructions of JUNV and MACV GPC. **a**, SDS-PAGE gel of purified wild-type (WT) or K33A_SSP_ JUNV GPC performed under reducing conditions visualized using a stain-free system. The experiment was performed twice, and a representative gel is shown. **b**, Micrograph of WT JUNV GPC and 2D class averages from 57,213 picked particles. Scale bar, 100 nm. The experiment was performed twice, and a representative gel is shown. **c**, Workflow used for cryo-EM data processing of K33A_SSP_ JUNV GPC. A representative micrograph is shown. Scale bar, 100 nm. Fourier shell correlation curves are shown. A threshold of 0.143 was used to estimate the resolution. Maps colored according to local resolution estimates were generated using ResMap^61^. **d**, SDS-PAGE gel of purified K33A_SSP_ MACV GPC performed under reducing conditions visualized using a stain-free system. The experiment was performed twice, and a representative gel is shown. **e**, Workflow used for cryo-EM data processing of K33A_SSP_ MACV GPC. A representative micrograph is shown. Scale bar, 100 nm. Fourier shell correlation curves, angular distribution of particles, and maps colored to local resolution are shown. A threshold 0.143 was used to estimate the resolution. Maps colored according to local resolution were generated using ResMap^61^. Source data

We generated and affinity purified full-length WT JUNV GPC (Fig. 1a) containing a tag from cell membranes. We observed bands for JUNV GP1 and GP2 by sodium dodecyl sulfate-polyacrylamide gel electrophoresis (SDS–PAGE) analysis, suggesting processing by SKI-1/S1P (Extended Data Fig. 1a). In vitrified samples of WT JUNV GPC visualized using cryo-EM, particles were too heterogeneous for structure determination, suggesting that they were a mixture of the pre- and post-fusion JUNV GPC or had been denatured during vitrification (Fig. 1b and Extended Data Fig. 1b).

**Extended Data Fig. 2:**
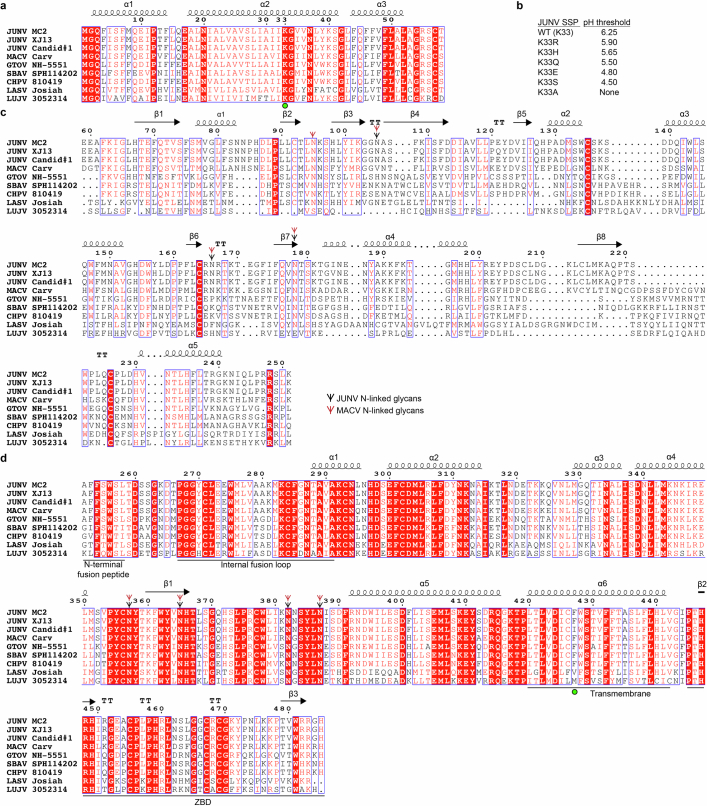
Sequence alignment of arenavirus GPCs. **a**, Sequence alignment of arenavirus SSP subunits. The green circle indicates the position of K33. **b**, Summary of pH thresholds for membrane fusion by JUNV GPC based on different substitutions of K33_SSP_ as determined by York et al. using cell-cell fusion assays^13^. **c**, Sequence alignment of arenavirus GP1 subunits. **d**, Sequence alignment of arenavirus GP2 subunits. The green circle indicates the position of F427_GP2_, the site of a substitution found in the JUNV vaccine strain Candid#1. Accession numbers for sequences used in the alignment are Junin virus (JUNV) (MC2 strain GenBank: BAA00964.2; XJ13 GenBank: ACO52428.1; Candid#1 strain GenBank: AAB65464.1), Machupo virus (MACV) (Carvallo GenBank: AAN05425.1), Guanarito virus (GTOV) (NH-5551 GenBank: AAN05423.1), Sabia virus (SBAV) (SPH114202 GenBank: AFA53089.1), Chapare virus (CHPV) (810419 GenBank: ABY87068.1), Lassa virus (LASV) (Josiah GenBank: AAA46283.1), and Lujo virus (LUJV) (3052314 GenBank: ACR56359.1). Numbering and secondary structure elements for JUNV GPC are shown. Panels were generated using ESPript 3.0.10 (ref. ^73^).

SSP residue K33 (K33_SSP_) is conserved in Old and New World arenaviruses (Extended Data Fig. 2a). It was previously shown that replacing K33_SSP_ with residues whose side chains have different sizes and charges alters the pH threshold for membrane fusion, and replacing it with an alanine (K33A_SSP_) completely abrogates GPC fusion activity in addition to reducing GP1 shedding (Extended Data Fig. 2b)^13,19^. We hypothesized that K33A_SSP_ stabilizes the prefusion conformation of GPC.

**Extended Data Fig. 3:**
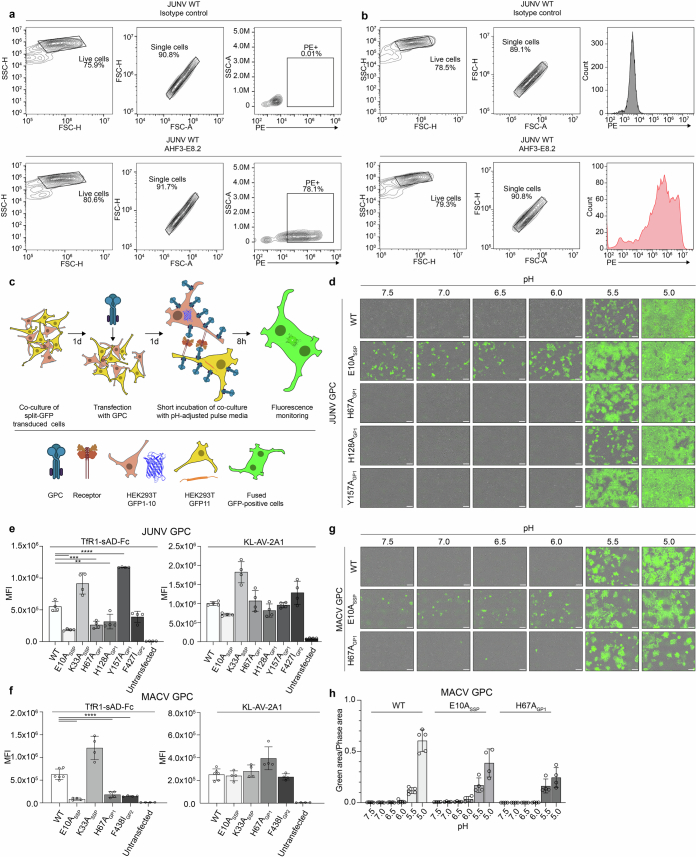
Flow cytometry gating scheme, cell-cell fusion assays, GP2 antibody staining, and effects of GPC mutations on fusion activity. **a**, Flow cytometry gating strategy for immunostaining experiments of GPC-transfected cells. PE: *R*-phycoerythrin. **b**, Flow cytometry gating strategy for immunostaining experiments evaluating cell surface expression of WT or mutant GPC constructs used in cell-cell fusion assays. **c**, Schematic describing the protocol used for split-GFP cell-cell fusion assays. Created with BioRender.com Mann, C. (2025) https://BioRender.com/imne67a. **d**,**g**, Representative images from split-GFP cell-cell fusion assays of HEK 293 T cells transiently transfected with WT or mutant JUNV GPC (**d**) and WT or mutant MACV GPC (**g**) with exposure of cells to pulse media at the indicated pHs. Images were obtained with a live-cell imager. Scale bar, 100 µm. For (**d**), data quantification is provided in Fig. 3j. For (**g**), quantification is provided in panel **h**. **e**,**f**, Results of immunostaining experiments of WT or mutant JUNV GPC (**e**) or WT or mutant MACV GPC (**f**). Staining was measured by flow cytometry using TfR1-sAD-Fc (a GP1-reactive reagent) or KL-AV-2A1 (a GP2-reactive antibody)^30^. Data are mean ± s.d. for experiments each performed in technical triplicate with the following numbers of independent experiments: for all samples in (**e**) (*n* = 4); for samples in (**f**), WT MACV (*n* = 6), all other samples (*n* = 4). One-way ANOVA with Dunnett’s multiple comparison test. ****P < 0.0001; ***P = 0.0004; **P = 0.0035 (**e**). ****P < 0.0001 (**f**). **h**, Quantification of data shown in **g**. Data are provided as GFP-positive area divided by total cell-covered area. Data are mean ± s.d. for experiments each performed in technical triplicate with the following numbers of independent experiments: WT (*n* = 5), E10A (*n* = 4), H67A (*n* = 4). Source data

To first test whether K33A_SSP_ would affect the antigenicity of JUNV GPC, we transfected cells with WT or K33A_SSP_ JUNV GPC and performed cell surface immunostaining experiments using GP1-reactive monoclonal antibodies. These included vaccine-elicited human antibodies (CR1-07, CR1-10 and CR1-28)^20^ and AHF-survivor-derived antibodies (AHF1-B7, AHF3-C5 and AHF3-E8.2). All antibodies bound cells transfected with WT or K33A_SSP_ JUNV GPC to similar levels (Fig. 1c,d and Extended Data Fig. 3a). An Fc-fusion protein that contains the soluble apical domain of a New World arenavirus rodent host TfR1 (TfR1-sAD-Fc)^21^ also bound cells transfected with WT or K33A_SSP_ JUNV GPC to comparable levels (Fig. 1c,d).

**Extended Data Fig. 4:**
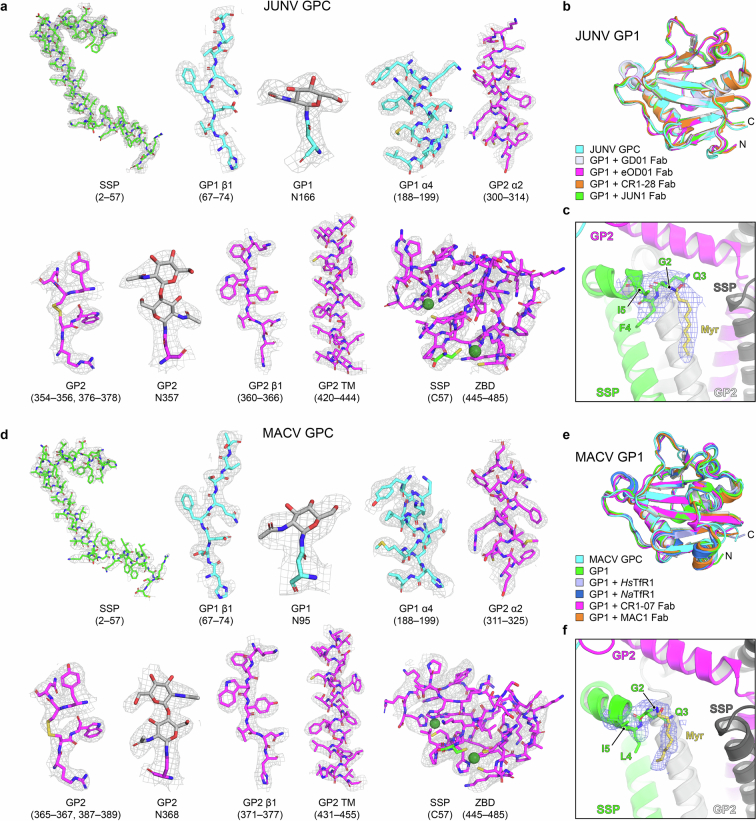
Sample cryo-EM density for JUNV and MACV GPC and comparisons with prior GP1 structures. **a**,**d**, Examples of cryo-EM density for segments of JUNV GPC (**a**) and MACV GPC (**d**). Segments of SSP, GP1, and GP2 are shown as sticks. Zinc ions are shown as green spheres. **b**,**e**, Superposition of JUNV GP1 (**b**) and MACV GP1 (**e**) as part of the GPC cryo-EM structures and previously determined X-ray crystal structures. For JUNV, these include structures of GP1 bound to the antigen-binding fragments (Fabs) of neutralizing antibodies GD01 (PDB: 5EN2)^22^, eOD01 (5NUZ)^24^, CR1-28 (5W1K)^20^, and JUN1 (7QU2)^23^. For MACV, these include structures of unliganded GP1 (PDB: 2WFO)^26^, GP1 bound to the ectodomain of human TfR1 (PDB: 3KAS)^27^, GP1 bound to a soluble form of the apical domain of *Neotoma albigula* (rodent ortholog) TfR1 (PDB: 6S9J)^21^, and GP1 bound to the Fabs of neutralizing antibodies CR1-07 (PDB: 5W1M)^20^ and MAC1 (PDB: 7QU1)^23^. N: N terminus; C: C terminus. **c**,**f**, Cryo-EM density associated with G2_SSP_ consistent with myristoylation. The myristoyl group (myr) was modeled in *Coot* and is shown here for illustrative purposes; it was not deposited in final models because it could not be modeled unambiguously. SSP residues 2–5 are shown as sticks.

Cryo-EM analysis of purified K33A_SSP_ JUNV GPC at the two-dimensional (2D) classification step revealed secondary structure features for the GPC ectodomain and membrane-spanning segments (Fig. 1b and Extended Data Fig. 1c). We obtained a 3.0 Å map of K33A_SSP_ JUNV GPC (Fig. 1e, Extended Data Fig. 4a and Supplementary Table 1). Superposition of GP1 as part of the GPC structure with previous crystal structures of JUNV GP1 (refs. ^20,22–24^) did not reveal conformational changes (Extended Data Fig. 4b). SSP residue G2 is myristoylated^12,25^. We observed density that is contiguous with G2_SSP_ and consistent with a myristoyl moiety at low map contour levels (Extended Data Fig. 4c).

### Cryo-EM structure of MACV GPC

**Fig. 2:**
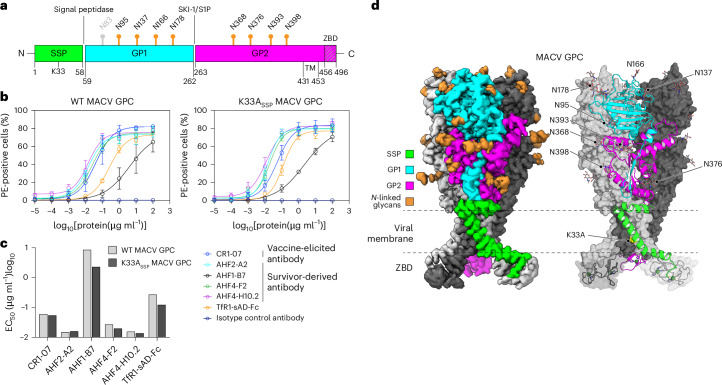
Structure of MACV GPC. **a**, Domain organization of MACV GPC. Sites of potential *N*-linked glycosylation are indicated. A putative glycan at GP1 N83, for which no density was observed, is shown in grey. Signal peptidase and SKI-1/S1P protease processing sites are shown. **b**, Cell surface immunostaining of HEK 293T cells transiently transfected with WT or K33A_SSP_ MACV GPC. Staining was performed using the indicated monoclonal antibodies or TfR1-sAD-Fc. Data are mean ± s.d. of 3 experiments performed in technical triplicate (*n* = 3 independent experiments). **c**, EC_50_ values for immunostaining experiments with WT or K33A_SSP_ MACV GPC shown in **b**. **d**, Cryo-EM map (left) and model (right) of K33A_SSP_ MACV GPC. For the model, one protomer is shown as a ribbon diagram and the others are shown as surfaces. The ZBDs found at the GP2 C termini are shown as ribbon diagrams with semi-transparent surfaces. Zinc ions are shown as green spheres. The positions of *N*-linked glycans, the K33A_SSP_ substitution and the viral membrane are indicated. Source data

The sequence of MACV GPC is 69% identical to that of JUNV GPC, and GP1 is the most diverse subunit (45% sequence identity) (Extended Data Fig. 2). We found that vaccine-elicited cross-reactive monoclonal antibody (CR1-07)^20^ and AHF-survivor-derived antibodies that cross-react with MACV GP1 (AHF2-A2, AHF1-B7, AHF4-F2, AHF4-H10.2) bound similarly to cells transfected with either WT or K33A_SSP_ MACV GPC (Fig. 2b,c). We affinity purified tagged MACV GPC and subjected samples to SDS–PAGE analysis, which revealed bands for GP1 and GP2 (Extended Data Fig. 1d). We obtained a 2.9 Å map of MACV GPC (Fig. 2d, Extended Data Figs. 1e and 4d, and Supplementary Table 1). Superposition of MACV GP1 from the GPC structure with previous crystal structures of MACV GP1 (refs. ^20,21,23,26,27^) did not reveal a conformational change (Extended Data Fig. 4e). Cryo-EM density for the myristoyl moiety on G2_SSP_ was observed at low map contour levels (Extended Data Fig. 4f).

### GPC trimerization mechanisms

**Fig. 3:**
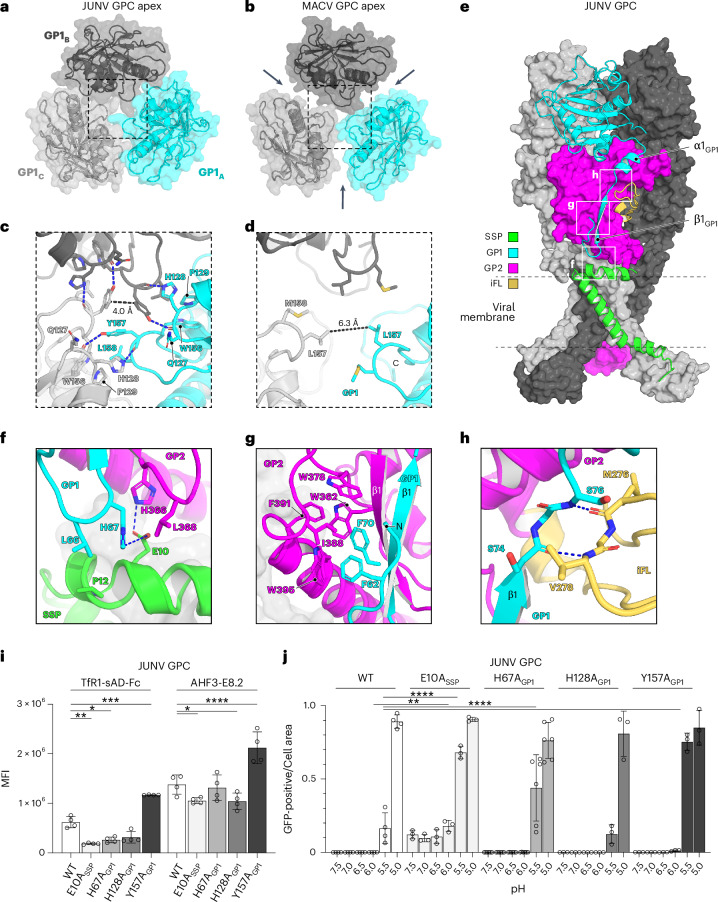
GPC intersubunit contacts. **a**,**b**, Top views of the JUNV (**a**) or MACV (**b**) GPC apices, showing organization of the three GP1 protomers (denoted A–C). In JUNV GPC, the GP1 subunits pack tightly against each other, while in MACV GPC, they are more widely spaced, as indicated by arrows. Dashed boxes provide orientation for the zoomed-in views provided in **c** and **d**. **c**,**d**, Zoomed-in view of GP1-GP1 contacts at the JUNV GPC apex (**c**) or MACV GPC apex (**d**). Residues that make interprotomer contacts are shown as sticks. Polar interactions are shown as blue dashed lines. **e**, Model of JUNV GPC providing orientation for panels **f**–**h**, as indicated by white boxes. Dashed lines represent the viral membrane. iFL, internal fusion loop. **f**–**h**, Zoomed-in view of intersubunit interactions occurring at the SSP-GP1-GP2 interface (**f**), the GP1-GP2 interface near the GP1 N-terminal hairpin loop (**g**) or at the GP1-GP2 iFL interface (**h**). Polar contacts are indicated as blue dashed lines. **i**, Results of immunostaining experiments measuring cell surface expression of WT or mutant JUNV GPC. Expression was measured by flow cytometry using either TfR1-sAD-Fc or AHF3-E8.2 for detection. Data are mean ± s.d. of 4 experiments, each performed in technical triplicate (*n* = 4). Two-way ANOVA with Dunnett’s multiple comparisons test (**i**). For TfR1-sAD-Fc: *P = 0.0225; **P = 0.0037; ***P = 0.002. For AHF3-E8.2: E10A *P = 0.0421; H128A *P = 0.0332; ****P = < 0.0001. **j**, Results of cell–cell fusion assays for WT or mutant JUNV GPC measured at different pHs. Cell–cell fusion was quantified using a split-GFP system and live-cell imaging for green fluorescence. Data are provided as GFP-positive area divided by total cell-covered area. See Extended Data Fig. 3c,d and Methods for additional details. Data are mean ± s.d. of experiments, each performed in technical triplicate with the following numbers of independent experiments: WT (*n* = 4); E10A, H128A and Y157A (*n* = 3); H67A (*n* = 6). Two-way ANOVA with Dunnett’s multiple comparisons test. **P = 0.0093, ****P < 0.0001. Source data

**Extended Data Fig. 5:**
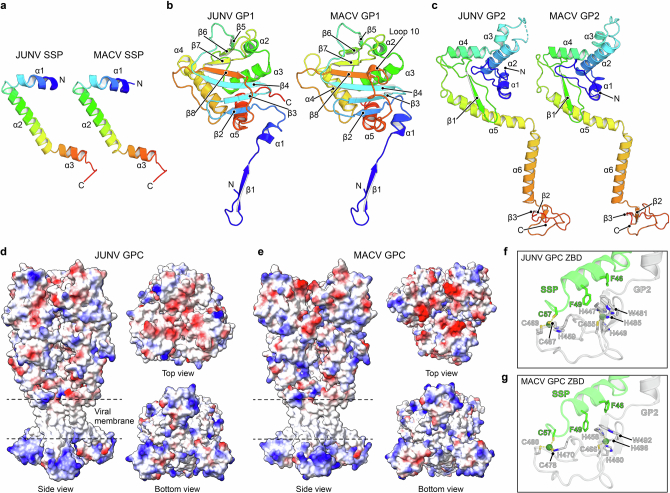
Secondary structure and electrostatic potentials of JUNV and MACV GPC subunits. **a**–**c**, Ribbon diagrams showing secondary structure elements in SSP (**a**), GP1 (**b**), and GP2 (**c**). The positions of N and C termini are also indicated. For MACV GP1, the C terminus is in the backplane and is therefore not indicated. **d**,**e**, Electrostatic potential of JUNV GPC (**d**) and MACV GPC (**e**) as determined using UCSF ChimeraX^62^. **f**,**g**, Zinc-binding domains (ZBDs) of JUNV GPC (**f**) and MACV GPC (**g**). Zinc ions are shown as green spheres. Residues involved in SSP-GP2 interactions or in metal coordination are shown as sticks.

The isolated SSP, GP1 and GP2 subunits of JUNV and MACV GPC are similar (Extended Data Fig. 5a–c); however, both GPCs differ in how their GP1 subunits are organized. The MACV GP1 subunits are slightly rotated outwards and clockwise compared with JUNV GP1 subunits when the trimers are observed from the top view, resulting in increased spacing between the subunits and giving the MACV GPC apex a more open appearance (Fig. 3a,b).

At the JUNV GPC apex, the side chains of Y157_GP1_ encircle the 3-fold axis of the trimer (Fig. 3c). Y157_GP1_, H128_GP1_ and the backbone carbonyl of Q127_GP1_ interact across the trimer interface (Fig. 3c). L158_GP1_ also makes hydrophobic contacts with P129_GP1_ and W156_GP1_ from adjacent protomers. In comparison, there are no GP1-GP1 contacts at the apex of MACV GPC (Fig. 3d).

### Intersubunit interactions

**Extended Data Fig. 6:**
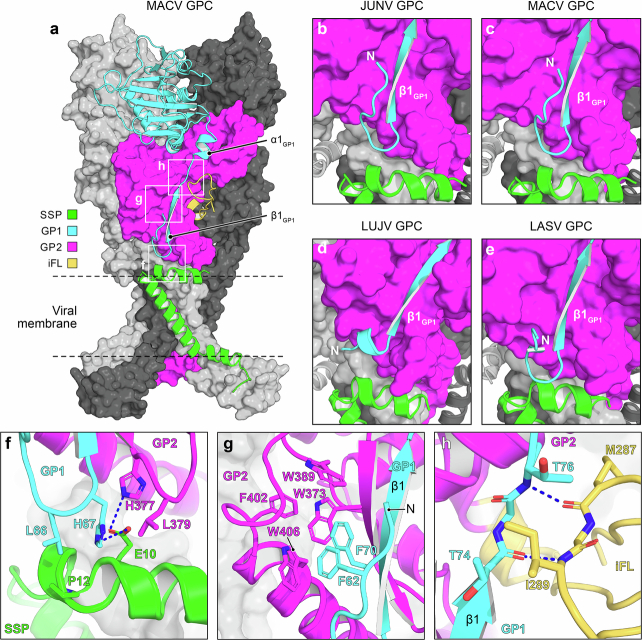
Organization of GP1 N-terminal region of arenavirus GPCs and MACV GPC inter-subunit interactions. **a**, Model of MACV GPC, providing orientation for panels **f**–**h**, as indicated by the white boxes. Dashed lines represent the position of the viral membrane**. b**–**e**, GP1 N-terminal region preceding the conserved β1 strand for JUNV GPC (**b**), MACV GPC (**c**), LUJV GPC (PDB: 8P4T)^29^ (**d**), and LASV GPC (PDB: 7PUY)^28^ (**e**). **f**–**h**, Views of inter-subunit interactions at the MACV GPC SSP/GP1/GP2 interface (**f**), the GP1-GP2 interface near the GP1 N-terminal hairpin loop (**g**), and the GP1-GP2 internal fusion loop (iFL) interface (**h**). Polar contacts are shown as blue dashes.

The N-terminal region of JUNV and MACV GP1 contains a short hairpin that is followed by a β-strand (β1) involved in an anti-parallel β-sheet with GP2 β1 (Fig. 3e and Extended Data Fig. 6a–c). This N-terminal hairpin is also in Lassa virus (LASV) GPC^28^, but not in Lujo virus (LUJV) GPC^29^, which has a short α-helix (Extended Data Fig. 6d,e). In JUNV GPC, H67_GP1_ is at the tip of the hairpin loop and contacts E10_SSP_ (Fig. 3f). E10_SSP_ is also contacted by H366_GP2_. The hairpin loop and β1 in JUNV GP1 include F62_GP1_ and F70_GP1_, which make non-polar contacts with a hydrophobic pocket on GP2 (Fig. 3g). In addition, the loop that connects GP1 β1 and α1 makes backbone-to-backbone contacts with residues in the GP2 internal fusion loop (Fig. 3h). Most of these intersubunit interactions are conserved in MACV GPC (Extended Data Fig. 6f–h).

### Organization of New World arenavirus GP1 C termini

**Fig. 4:**
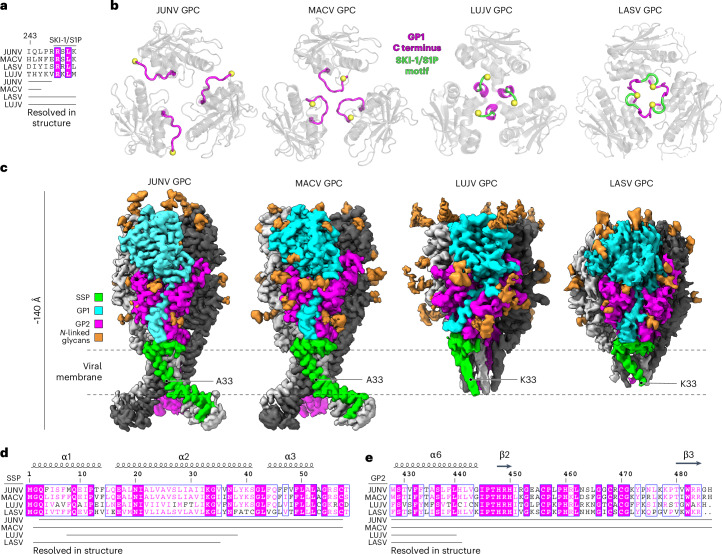
Comparison of GP1 C termini and membrane-spanning segments in GPC structures. **a**, Partial sequence alignment of the GP1 C termini in GPC structures described here and in previous studies^28,29^. The SKI-1/S1P protease processing site is also indicated. **b**, Top-down views of the GPC apices for MACV, JUNV, LUJV (PDB: 8P4T)^29^ or LASV (PDB: 7PUY)^28^. The yellow sphere indicates the position of the most C-terminal residue. Residues forming the SKI-1/S1P site, which are resolved in the LUJV and LASV GPC structures, are shown in green. **c**, Comparison of cryo-EM maps for JUNV, MACV, LUJV (EMD-17428)^29^ or LASV (EMD-13662)^28^ GPC. The location of SSP residue 33 in each model is indicated. **d**,**e**, SSP (**d**) or GP2 (**e**) sequence alignment of the indicated New World and Old World arenaviruses generated using ESPript (3.0)^73^. Residues that are resolved in the GPC cryo-EM structures reported here, or in LUJV GPC (PDB: 8P4T)^29^ and LASV GPC (PDB: 7PUY)^28^, are indicated.

For LASV^28^ and LUJV GPC^29^, SKI-1/S1P motif residues make trimerization contacts at the GPC apex (Fig. 4a,b). The SKI-1/S1P motif in LASV GPC forms a receptor-binding surface that interacts with matriglycan^28^. In maps of JUNV and MACV GPC, the GP1 SKI-1/S1P motif residues could not be resolved, suggesting that these regions are flexible (Fig. 4a). Furthermore, the visualized portions of the JUNV and MACV GP1 C termini are not at the trimer apices. The JUNV GP1 C termini are in clefts formed by the GP1 subunits of adjacent protomers and GP2, and project away from the 3-fold axis of trimeric GPC (Fig. 4b). The MACV GP1 C termini point towards the 3-fold axis of GPC but are far from the apex (Fig. 4b). Differences in positioning may explain the lack of sequence conservation in the GP1 C termini among arenaviruses (Fig. 4a).

### GP2 architecture and zinc-binding domain (ZBD) assembly

In cryo-EM structures of K33A_SSP_ JUNV and MACV GPC, unlike in cryo-EM structures of LASV and LUJV GPC^28,29^, we could resolve the full TM segments for SSP and GP2, and the ZBD (Fig. 4c–e). The ZBD surface that would face the inner leaflet of the viral membrane is positively charged, which may promote interactions with phospholipid head groups (Extended Data Fig. 5d,e). Map features were poorer in the ZBD regions, suggestive of flexibility (Extended Data Figs. 1c,e and 4a,d). The C-terminal portions of SSP form a helix (α3) that would be partially embedded in the viral membrane. Residues C57_SSP_ and H459_GP2_, C467_GP2_ and C469_GP2_ coordinate one zinc ion, and the other zinc ion is coordinated by H447_GP2_, H449_GP2_, C455_GP2_ and H485_GP2_ (Extended Data Fig. 5f). ZBD organization is generally conserved in MACV GPC (Extended Data Fig. 5g).

### Intramembranous interactions in GPC

**Fig. 5:**
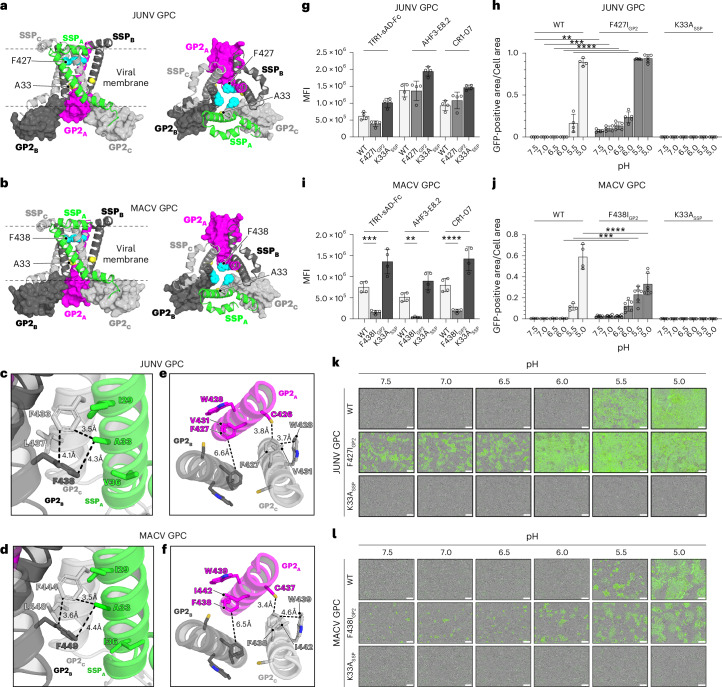
Effects of substitutions in GPC TM regions on pH-dependent fusion. **a**,**b**, Top (left) and side (right) views of the JUNV GPC (**a**) or MACV GPC (**b**) showing the segments that would be positioned in or near the viral membrane. JUNV GPC F427_GP2_ and A33_SSP_, and MACV GPC F438_GP2_ and A33_SSP_ are shown as spheres. **c**,**d**, Zoomed-in views showing residues that are near A33_SSP_ in structures of JUNV GPC (**c**) or MACV GPC (**d**). A33_SSP_ lines a hydrophobic pocket that is formed by residues from the same SSP protomer and the GP2 TM (α6) helices of two adjacent protomers. Relevant distances are shown as black dashed lines. **e**,**f**, JUNV (**e**) or MACV (**f**) GP2 TM helices showing interactions between F427 (JUNV) or F438 (MACV) with neighbouring residues. Relevant distances are shown as black dashed lines. **g**,**i**, Cell surface staining of JUNV GPC and MACV GPC constructs transiently transfected in HEK 293T cells with immunostaining performed using TfR1-sAD-Fc or the indicated GP1-reactive antibodies. Data are mean ± s.d. of 4 experiments, each performed in technical triplicate (*n* = 4). **h**,**j**, Data from split-GFP cell–cell fusion assays of WT or mutant JUNV and MACV GPC in transiently transfected HEK 293T cells. Cell–cell fusion was quantified using a split-GFP system and live-cell imaging for green fluorescence. Data are provided as the GFP-positive area divided by total cell-covered area. See Extended Data Fig. 3c and Methods for additional details. Data are mean ± s.d. of the following number of independent experiments: WT (*n* = 4), F427I and K33A (*n* = 5) (**h**); WT (*n* = 4), F438I (*n* = 6), K33A (*n* = 5) (**j**). Two-way ANOVA with Dunnett’s multiple comparisons test. **P = 0.0044, ***P = 0.0001 and ****P < 0.0001 (**h**). ***P = 0.0002 and ****P < 0.0001 (**j**). **k**,**l**, Representative images from split-GFP cell–cell fusion assays of HEK 293T cells transiently transfected with WT or mutant JUNV GPC with exposure to pulse media at the indicated pH values. Images were obtained with a live-cell imager. Scale bar, 100 µm. Source data

In K33A_SSP_ JUNV and MACV GPC, A33_SSP_ lines a hydrophobic pocket that would be in the viral membrane and is formed by portions of the TM α-helix of SSP (α2) and two GP2 TM α-helices (α6) (Fig. 5a–d). The pocket in JUNV GPC is formed by F433_GP2_, L437_GP2_ and F438_GP2_ (Fig. 5c). The analogous pocket in MACV GPC is formed by F444_GP2_, L448_GP2_ and F449_GP2_ (Fig. 5d).

Substitution F427I_GP2_ is the major determinant of Candid#1 attenuation^16,17^. Introducing the analogous substitution into MACV GPC (F438I_GP2_) attenuates MACV; however, the resultant virus reverts to WT in infected mice^18^. JUNV F427_GP2_ is in GP2 α6 and is conserved in New World arenaviruses (Extended Data Fig. 2d). In JUNV, the side chain of F427_GP2_ interacts with the adjacent W428_GP2_ and V431_GP2_ from the same GP2 α6 helix, and with C426_GP2_ from the neighbouring GP2 α6 helix (Fig. 5e). The contacts that MACV F438_GP2_ makes with neighbouring residues in the GP2 α6 helices are similar (Fig. 5f). The structures thus suggest that F427I_GP2_ (JUNV) and F438I_GP2_ (MACV) disrupt local hydrophobic interactions in the GP2 TM helices and destabilize GPC.

### Cell–cell fusion assays to dissect effects on membrane fusion

We next used split-GFP cell–cell fusion assays to study the role of specific contacts observed in the structures on pH-dependent membrane fusion (Extended Data Fig. 3c). We confirmed cell surface expression of WT, F427I_GP2_ or K33A_SSP_ JUNV GPC using immunostaining with TfR1-sAD-Fc or antibodies that cross-react between JUNV and MACV GP1. We also used an anti-GP2 murine non-neutralizing antibody that cross-reacts with arenavirus GPCs (KL-AV-2A1)^30^. All constructs were expressed at the cell surface and contained GP1 and GP2 (Fig. 5g and Extended Data Fig. 3b,e). For cells transfected with WT JUNV GPC, we observed cell–cell fusion at pH 5.0 and 5.5, consistent with the optimal pH of JUNV GPC-mediated membrane fusion (Fig. 5h,k)^13,19,31^. The K33A_SSP_ mutant, as expected, lacked activity at all pH values^13^. We observed more cell–cell fusion at pH 5.5 and 6.0 for the F427I_GP2_ mutant and even observed some fusion at near physiological pH (7.5) (Fig. 5h,k).

We could detect cell surface expression of WT, F438I_GP2_ and K33A_SSP_ MACV GPC (Fig. 5i and Extended Data Fig. 3f). Interestingly, staining of F438I_GP2_ with GP1-reactive reagents (TfR1-sAD-Fc, AHF3-E8.2 or CR1-07) was poor despite retained staining by GP2-reactive antibody KL-AV-2A1. These findings suggest that poor staining of F438I_GP2_ by GP1-reactive reagents could be explained by increased GP1 shedding.

We observed no cell–cell fusion for K33A_SSP_ MACV GPC (Fig. 5j,l). Wild-type MACV GPC caused membrane fusion at pH 5.0 and 5.5. Although the phenotype was milder, probably due to increased GP1 shedding, F438I_GP2_ MACV GPC had activity at pH 6.0, with detectable activity up to pH 7.5; however, the increase in membrane fusion did not reach statistical significance at pH values of 6.5–7.5. Taken together, the structures and cell–cell fusion assay results suggest that the F427I_GP2_ (JUNV) and F438I_GP2_ (MACV) substitutions weaken interactions between GP2 TM helices to destabilize the GPC complex, resulting in membrane fusion at neutral pH.

We also examined substitutions that would disrupt GP1 trimerization contacts at the JUNV GPC apex (H128A_GP1_ and Y157A_GP1_) (Fig. 3c) and those that would disrupt GP1/GP2/SSP interactions near the viral membrane (E10A_SSP_ and H67A_GP1_) (Fig. 3f). We confirmed GPC expression using immunostaining with TfR1-sAD-Fc and KL-AV-2A1 (Fig. 3i and Extended Data Fig. 3e). In cell–cell fusion assays, the H128A_GP1_ JUNV GPC apex mutant behaved similarly to the WT protein (Fig. 3j and Extended Data Fig. 3d). For the Y157A_GP1_ JUNV GPC apex mutant, we observed more fusion activity at pH 5.5 but no fusion at higher pH values, suggesting that disruption of this apex trimerization contact only modestly destabilizes GPC. The H67A_GP1_ JUNV GPC mutant, which would disrupt interaction between H67_GP1_ and E10_SSP_, had a mild effect compared with WT JUNV GPC that was only observed at pH 5.5. However, the E10A_SSP_ mutant, which would disrupt SSP interactions with both H67_GP1_ and H366_GP2_, was more active at pH 5.5 compared with WT GPC, with cell–cell fusion observed at pH values up to 7.5.

The MACV GPC H67A_GP1_ mutant, which would disrupt interactions between H67A_GP1_ with E10_SSP,_ and the E10A_SSP_ mutant, which would disrupt interactions between H67_GP1_ and H377_GP2_, were poorly detected by TfR1-sAD-Fc despite retaining immunostaining by KL-AV-2A1 (Extended Data Figs. 3f and 6f). These findings suggest that the MACV GPC H67A_GP1_ and E10A_SSP_ mutations increased GP1 shedding, thus impairing GPC function. We only observed pH-dependent membrane fusion at pH values of 5.0 and 5.5 for the H67A_GP1_ mutant, which is similar to WT MACV GPC (Extended Data Fig. 3g,h). However, the E10A_SSP_ MACV GPC had some, albeit very weak, activity at pH values ranging from 6.0 to 7.5.

### GPC SSP–TM interactions in modelled membranes

**Fig. 6:**
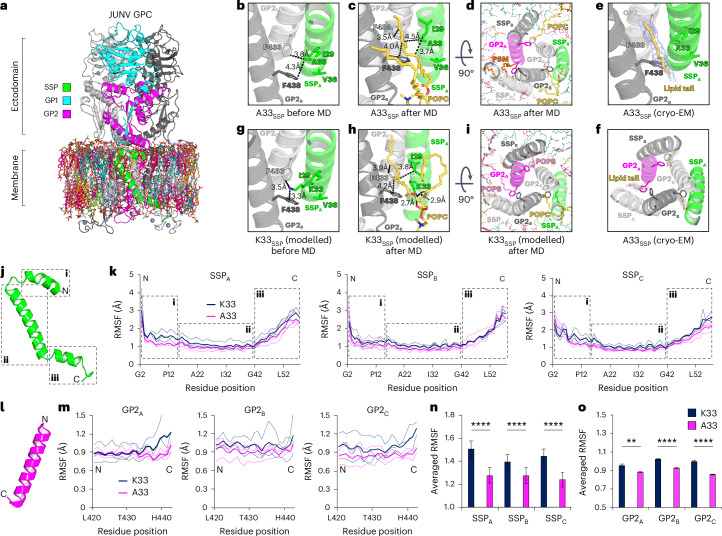
Predicted lipid interactions and K33A_SSP_ stabilization of JUNV GPC TM regions. **a**, Membrane-embedded JUNV GPC used for molecular dynamics (MD) simulations. MD simulations were performed in triplicate (*n* = 3). See Extended Data Fig. 7 for additional information. **b**, JUNV GPC A33_SSP_ and neighbouring residues in the cryo-EM structure. Relevant distances are shown as dashed lines. **c**, JUNV GPC A33_SSP_ in a representative frame focused on a pocket at the SSP-GP2 interface at the end of the MD simulation. POPC, phosphatidylcholine. **d**, Top view of the JUNV GPC A33_SSP_ GPC TM helices in a representative frame at the end of the simulation. In addition to the POPC tail occupying one of the pockets, the other pockets are occupied by a second POPC and a palmitoylsphingomyelin (PSM) tail. **e**,**f**, Structure of A33_SSP_ JUNV GPC showing lipid-like cryo-EM density occupying the pocket in a side (**e**) or top view (**f**). Part of a lipid tail is modelled (yellow sticks) for illustrative purposes. **g**, WT JUNV GPC (K33_SSP_, modelled in silico) and neighbouring hydrophobic residues. Relevant distances are shown as dashed lines. **h**, JUNV GPC K33_SSP_ in a representative frame focused on the pocket at the end of the MD simulation. The pocket is occupied by a POPC lipid tail. **i**, Top view of the JUNV GPC K33_SSP_ TM helices in a representative frame at the end of the simulation. In addition to the POPC lipid tail occupying one of the pockets, the two other pockets are occupied by 1-palmitoyl-2-oleoyl-*sn*-glycero-3-phosphoserine (POPS) lipid tails. **j**, JUNV GPC SSP with dashed boxes serving as points of reference for plots shown in **k**. **k**, RMSF plots for the SSP chains. **l**, GP2 TM α-helix (residues 420–442). **m**, RMSF plots for the GP2 TM (α6) helices (residues 420–442). **n**,**o**, Mean RMSF values during MD simulations for the SSP protomers (**n**) or the GP2 TM α-helices (**o**) plotted for the WT (K33_SSP_) and A33_SSP_ JUNV GPC. Error bars represent standard errors. In **n** and **o**, comparison between two groups was performed using an unpaired, two-tailed Student’s *t*-test. **P = 0.003, ****P < 0.0001. Source data

**Extended Data Fig. 7:**
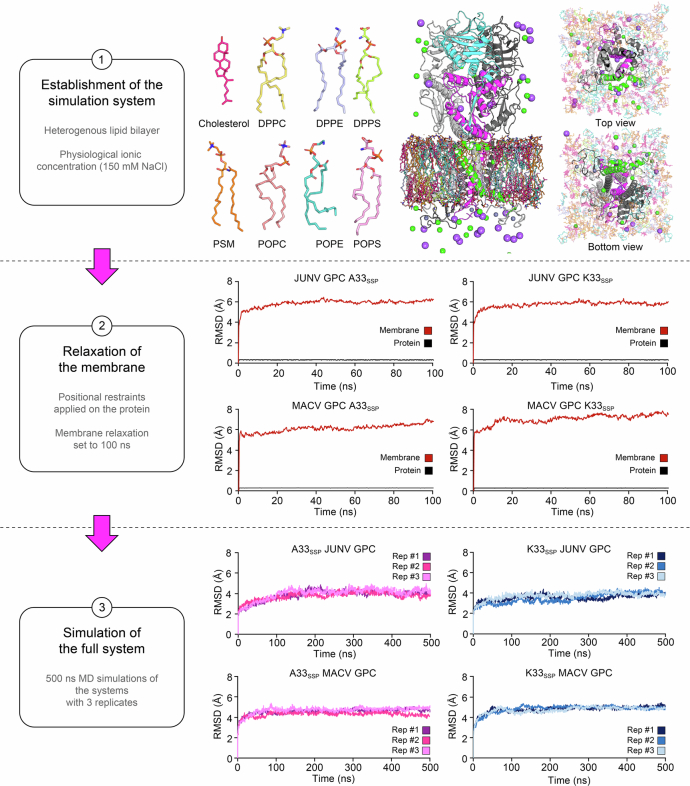
Molecular dynamics simulation workflow. Workflow for molecular dynamics simulation of membrane-embedded JUNV and MACV GPC. Membrane-embedded JUNV GPC is shown, with Na^+^ ions shown as purple spheres and Cl^−^ ions shown as green spheres. Abbreviations are as follows: DPPC, 1,2-dipalmitoyl-sn-glycero-3-phosphocholine; DPPE, 1,2-dipalmitoyl-sn-glycero-3-phosphoethanolamine; DPPS, 1,2-dipalmitoyl-sn-glycero-3-phospho-L-serine; PSM, palmitoylsphingomyelin; POPC, 1-palmitoyl-2-oleoyl-sn-glycero-3-phosphocholine; POPE, 1-palmitoyl-2-oleoyl-sn-glycero-3-phosphoethanolamine; POPS, 1-palmitoyl-2-oleoyl-sn-glycero-3-phosphoserine. RMSD: root-mean-square deviation. See Methods and Supplementary Table 3 for additional information. Source data

**Extended Data Fig. 8:**
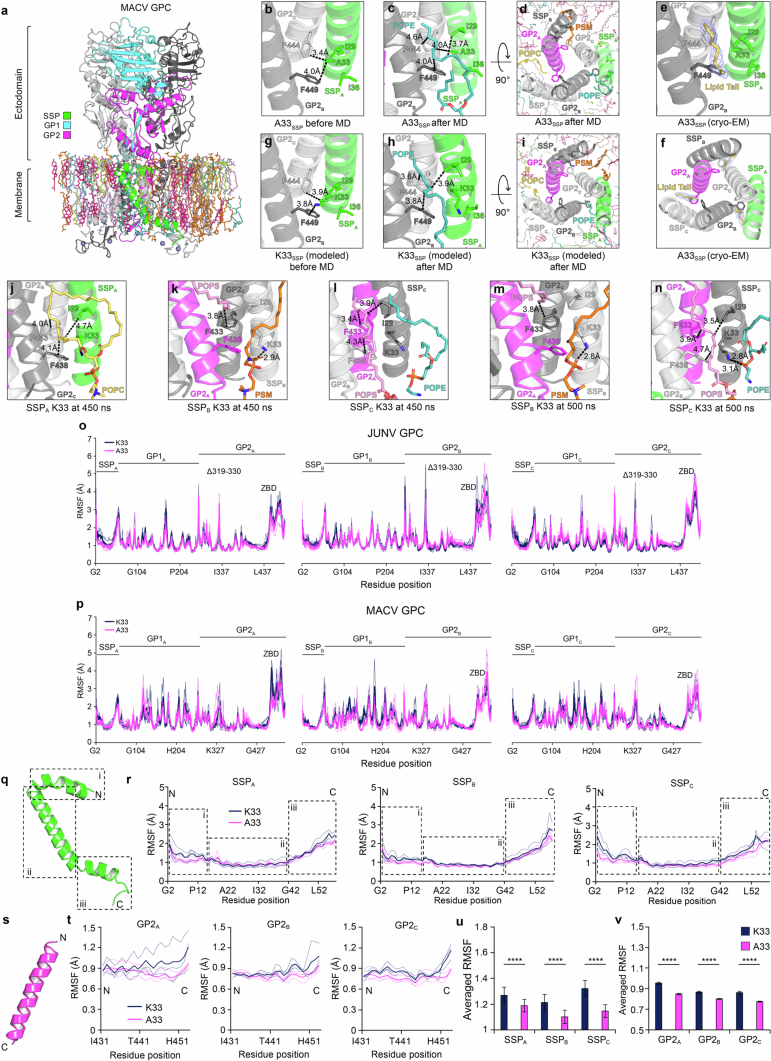
MACV GPC MD simulations and JUNV GPC K33 position during MD simulations. **a**, Membrane-embedded MACV GPC used for MD simulations performed in triplicate (*n* = 3). **b**, A33_SSP_ in the MACV GPC structure. Relevant distances are shown as dashed lines. **c**, A hydrophobic pocket near A33_SSP_ is occupied by a phosphatidylethanolamine (POPE) lipid tail at the end of the simulation. A representative frame is shown. **d**, Top view of GPC TM helices at the end of the MD simulation. In addition to the POPE tail occupying one of the pockets, the other pockets are occupied by a palmitoylsphingomyelin (PSM) tail and a phosphatidylcholine (POPC) tail partially occupies another pocket. **e**,**f**, Structure of A33_SSP_ MACV GPC showing lipid-like cryo-EM density occupying the pocket in a side view (**e**) or a top view (**f**). Part of a lipid tail is modeled (yellow sticks). **g**, WT MACV GPC (K33_SSP_, modeled *in silico*) and neighboring residues. **h**,**i**, Representative frames after MD simulation of WT (K33_SSP_) MACV GPC. A POPE tail is in the pocket. A side view (**h**) or a top view showing the three binding sites (**i**) are provided. **j**–**n**, Frames from the MD simulations showing the lack of a conserved contacts K33_SSP_. K33_SSP_ from different SSP protomers are shown. See Fig. 6h for a related frame (SSP_A_ at 500 ns). **o,p**, RMSF curves for MD simulations of A33_SSP_ (cryo-EM structure) of K33_SSP_ (mutated *in silico*) for JUNV (**u**) or MACV (**v**) GPC. Three thin lines represent individual MD runs, and a thick line represents averaged RMSF values. **q**, MACV GPC SSP with dashed boxes as reference for the RMSF plots shown in (**k**). **r**, RMSF plots for the three MACV GPC SSP chains. **s**, GP2 TM (α6) TM helix (residues 430–456). **t**, RMSF plots for the three GP2 TM α-helices. **u**,**v**, Mean RMSF values calculated during MD simulations for the SSP protomers (**n**) or the GP2 TM helices (**o**). In (**n**) and (**o**), comparison between two groups was performed using an unpaired, two-tailed Student’s t-test. Error bars represent standard errors. ****P < 0.0001. Source data

We next used molecular dynamics (MD) simulations to study how GPC may interact with lipids. We used heterogeneous lipid bilayers with ratios based on lipidomic data from other viruses (Fig. 6a, Extended Data Figs. 7 and 8a, and Supplementary Table 2)^32–34^. Following MD simulations with the A33_SSP_ JUNV and MACV GPC cryo-EM structures, we observed that lipid tails occupy the hydrophobic pocket that abuts A33_SSP_ (Fig. 6b–d and Extended Data Fig. 8b–d). The potential lipid binding sites were occupied with variable occupancy in different MD replicates (Supplementary Table 3). Supporting the results of the MD simulations, examination of the cryo-EM maps for A33_SSP_ JUNV and MACV GPC revealed features consistent with aliphatic chains at those sites (Fig. 6e,f and Extended Data Fig. 8e,f). This observation suggests that either detergent molecules or lipids carried over from purification of the protein can occupy the sites.

We next generated models of JUNV and MACV GPC containing the WT K33_SSP_ by introducing the substitution in silico. Before the MD simulation, the side chain of K33_SSP_ would be positioned unfavourably, occupying the hydrophobic pocket (Fig. 6g and Extended Data Fig. 8g). Simulations with WT JUNV and MACV GPC again revealed instances in which a lipid tail occupied the pocket, with the K33_SSP_ side chain in no preferred orientation (Fig. 6h,i and Extended Data Fig. 8h–n).

We used root-mean-square-fluctuation (RMSF) calculations to compare structural fluctuations in A33_SSP_ JUNV or MACV GPC, or K33_SSP_ JUNV or MACV GPC (Extended Data Fig. 8o,p and Supplementary Table 2). We observed that the K33A_SSP_ substitution decreases structural fluctuations in the SSP and GP2 TM α helices of both GPCs (Fig. 6j–o and Extended Data Fig. 8q–v).

Our findings suggest that the K33A_SSP_ substitution stabilizes the GPC TM regions. Conversely, they suggest that K33_SSP_ is critical for the intrinsic metastability of arenavirus GPCs.

## Discussion

The JUNV Candid#1 vaccine relies primarily on a GP2 TM substitution (F427I_GP2_) for its attenuation. There have been conflicting reports about the effects of the F427I_GP2_ substitution. One study observed that F427I_GP2_ does not alter the pH threshold for membrane fusion^35^. Another study found that F427I_GP2_ caused pH-dependent membrane fusion to be observed at neutral pH^31^. Our mutational analyses suggest that the F427I_GP2_ mutation modifies hydrophobic interactions between GP2 TM helices and destabilizes GPC to alter pH-dependent membrane fusion (Supplementary Table 4). We also found the analogous F438I_GP2_ mutation in MACV GPC results in cell–cell fusion at neutral pH. Interestingly, MACV GPC F438I_GP2_ was poorly stained by GP1-reactive reagents despite retained staining for GP2, suggesting that this substitution increased GP1 shedding (Fig. 5i and Extended Data Fig. 3f). The effect of the F427I_GP2_ mutation on JUNV GPC GP1 shedding seemed milder (for example, comparing WT and F427I_GP2_ JUNV GPC in Fig. 5g). Thus, the F438I_GP2_ substitution may have a higher fitness cost in MACV GPC, potentially explaining why the revertant mutation arises when mice are infected with recombinant MACV containing F438I_GP2_ (ref. ^18^).

A previous study showed that the K33S_SSP_ substitution substantially decreases, but does not completely ablate, JUNV GPC pH-dependent membrane fusion activity^35^. The smaller serine residue would be more favourable than a lysine in the viral membrane but would be less favourable than the alanine, explaining why K33S_SSP_ JUNV GPC still has some membrane fusion activity. Interestingly, JUNV GPC containing both the K33S_SSP_ and F427I_GP2_ substitutions has a pH-dependency profile that is closer to that of WT JUNV GPC, suggesting epistatic interactions between these residues^35^. Considering that K33_SSP_ and F427_GP2_ are far from each other in the JUNV GPC structure, a loss of stability within one part of the GPC TM region can be compensated by gains in stability elsewhere in the TM region.

Two previous studies proposed that the SSP N terminus is on the cytoplasmic side of the membrane^36,37^. However, all structural data so far^28,29^, including structures reported here, suggest that the SSP N terminus is on the same side of the GPC ectodomain with respect to membranes. In addition to confirming involvement of C57_SSP_ in zinc ion coordination as predicted in previous biochemical and NMR studies^38,39^, the JUNV GPC structure also reveals hydrophobic interactions between residues F49_SSP_ and W481_GP2_ (Extended Data Fig. 5f). Interestingly, both residues are conserved in Old and New World arenaviruses (Extended Data Fig. 2a,d), and F49_SSP_ is part of a conserved ‘FLLL’ sorting signal in SSP that is important for GPC maturation and trafficking^40^.

SSP and the GP2 membrane-proximal regions and TM segments probably contribute most of the energy required to stabilize GPC in the prefusion conformation. This property may explain why the soluble ectodomain of LASV GPC, comprising only GP1 and GP2, is usually monomeric in solution without the addition of a trimerization domain at the GP2 C terminus or an antibody to induce trimerization^41,42^. Similarly, the lymphocytic choriomeningitis virus (LCMV) GPC ectodomain is not a trimer in solution and requires addition of a trimerization domain^43,44^.

**Extended Data Fig. 9:**
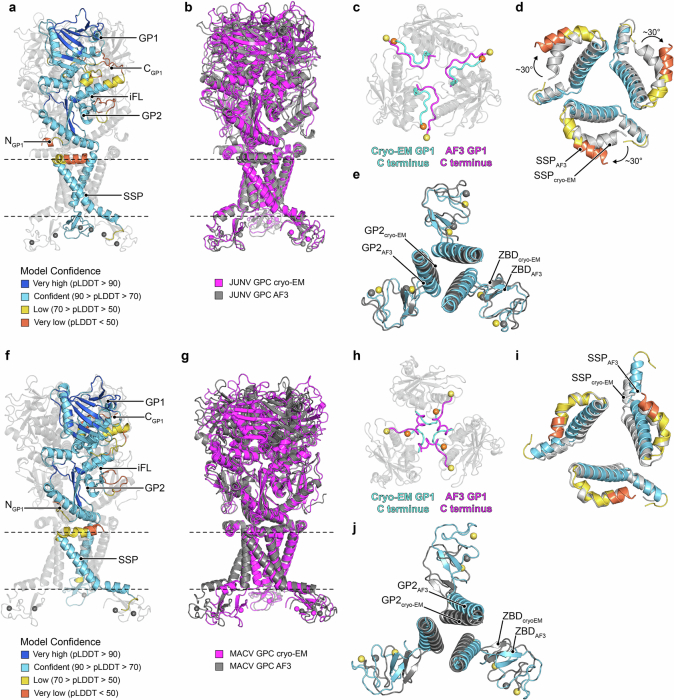
Comparison of JUNV and MACV GPC cryo-EM structures and AlphaFold 3 predictions. **a**,**f**, AlphaFold 3^45^ models of K33A_SSP_ JUNV GPC (**a**) and K33A_SSP_ MACV GPC (**f**), with a single protomer colored according to the pLDDT confidence metrics. Dashed lines represent the relative position of the viral membrane. **b**,**g**, Structural superposition of the K33A_SSP_ JUNV GPC AF3 model with the K33A_SSP_ JUNV GPC cryo-EM structure (**b**) and the K33A_SSP_ MACV GPC AF3 model with the K33A_SSP_ MACV GPC cryo-EM structure (**g**). **c**,**h**, Top views of the JUNV GPC (**c**) and MACV GPC (**h**) apex comparing the relative trajectory of the GP1 C terminus between the AF3 predicted model and cryo-EM structures. The yellow and orange spheres indicate the position of the most C-terminal residues. Only the GP1 domains from the cryo-EM structure are shown, and GP1 domains from the AF3 models are omitted for clarity. **d**,**i**, Top views of structural superpositions of the SSP subunits of JUNV GPC (**d**) and MACV GPC (**i**) derived from AF3 predictions and cryo-EM structures. AF3 models are colored using the pLDDT scheme shown in (**a**), and cryo-EM structures are shown in gray. **e**,**j**, Top views of GP2 TM and cytosolic segments from the superposed JUNV GPC (**e**) and MACV GPC (**j**) AF3-predicted models and cryo-EM structures. AF3 models are colored using the pLDDT scheme shown in (**a**) and cryo-EM structures are shown in gray. Zinc ions are shown as spheres. N: N terminus; C: C terminus; iFL: internal fusion loop.

To facilitate structural interpretation and model building into cryo-EM maps, we used AlphaFold 3 (AF3)-predicted models^45^. When comparing the AF3 models to the cryo-EM structures, although AF3 could predict general features of GPC assembly, there were differences in the predicted positions of SSP α1 helix, GP1 C termini, GP2 TM helices and the angles of the ZBDs with respect to the GPC ectodomain (Extended Data Fig. 9).

**Extended Data Fig. 10:**
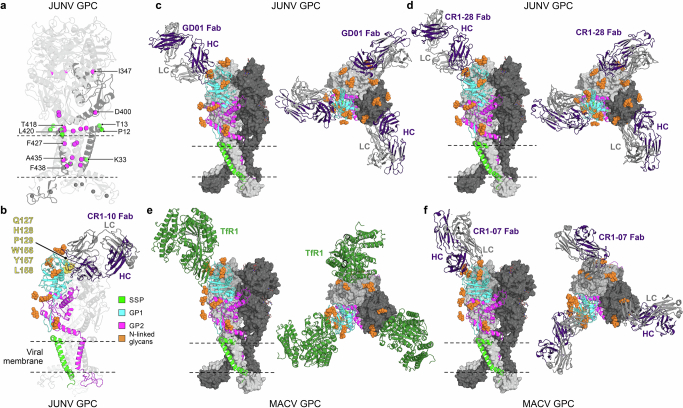
Inhibitor susceptibility determinants and modeling of neutralizing antibody- and receptor-bound JUNV and MACV GPC structures. **a**, JUNV GPC model indicating sites of substitutions previously shown to result in resistance to ST-193 or ST-294, two chemically unrelated small molecule inhibitors of pH-dependent membrane fusion that target SSP-GP2 interactions to inhibit membrane fusion^19,47,48,54^. Cα atoms at the location of substituted residues are shown as either green (SSP) or magenta (GP2) spheres for one of the SSP and GP2 protomers. Dashed lines indicate the relative position of the viral membrane. **b**, Superposition of JUNV GPC with the crystal structure of JUNV GP1 bound to the Fab of a non-neutralizing antibody, CR1-10 (5W1K)^20^. Residues that contact each other during trimerization at the GP1 apex are shown as yellow spheres and indicated (see also Fig. 3c). These residues are also within the footprint of CR1-10. **c**,**d**, Superposition of JUNV GPC with the crystal structure of JUNV GP1 bound to Fabs from neutralizing antibodies GD01 (PDB: 5EN2)^20^ (**c**) or CR1-28 (PDB: 5W1K)^20^ (**d**). **e**,**f**, Superposition of MACV GPC with the crystal structure of MACV GP1 bound to a monomer of the TfR1 ectodomain (PDB: 3KAS)^27^ (**e**) or to the Fab of neutralizing CR1-07 (PDB: 5W1M)^20^ (**f**).

The SSP-GP2 interface is targeted by small-molecule membrane fusion inhibitors^19,46–54^. Mutations that modulate drug sensitivity cluster in or near the GPC TM region (Extended Data Fig. 10a and Supplementary Table 5). We propose that small-molecule inhibitors of membrane fusion probably act like the K33A_SSP_ substitution to stabilize the SSP-GP2 interface, and that mutations that alter drug susceptibility may modify TM segment packing in addition to perturbing small-molecule binding.

Residue Y157_GP1_ makes important contacts at the apex of JUNV GPC but is not conserved among JUNV strains. Sequence variation in this GP1 region suggests that it may be under immune pressure from antibodies. The GP1 residues that make trimerization contacts at the apex of JUNV GPC are in the epitope for CR1-10, a non-neutralizing human antibody that binds a surface that would be occluded on an assembled GPC trimer (Extended Data Fig. 10b)^20^. Presumably, antibodies in arenavirus rodent hosts may also be binding this site and exerting selective pressure. The Y157A_GP1_ substitution has only a modest impact on JUNV GPC pH-dependent membrane fusion activity, suggesting that the virus can tolerate mutations at the GPC apex without incurring too large of a cost on viral fitness.

Superposing structures of MACV GP1 bound to TfR1 (ref. ^27^), or JUNV or MACV bound to neutralizing antibodies^20^, suggests that receptor and antibody-binding sites would be accessible despite differences in the rotation and openness of the GP1 trimers (Extended Data Fig. 10c–f). Although neutralizing antibodies targeting quaternary epitopes that involve GP1/GP2 or the trimer apex have been described for Old World arenaviruses LASV and LCMV^41–43,55–57^, such antibodies have not yet been described for New World arenaviruses. The structures reported here could facilitate the engineering of prefusion-stabilized New World arenavirus GPCs to accelerate the discovery of such antibodies. In addition, GPC containing the K33A_SSP_ substitution could be an attractive approach to developing prefusion-stabilized mRNA-based vaccines.

## Methods

### Inclusion and ethics statement

Monoclonal antibodies from convalescent survivors of Argentine haemorrhagic fever were obtained under Boston Children’s Hospital IRB protocol IRB-P00007578 and Harvard Medical School IRB protocol IRB19-1112. Written informed consent was obtained for the study.

### Cells

We maintained HEK 293T (human embryonic kidney, ATCC CRL-1268) cells in Dulbecco’s modified Eagle’s medium (DMEM; Gibco, 11995-073) supplemented with 10% (v/v) fetal bovine serum (FBS) at 37 °C and 8% CO_2_. We maintained Expi293F cells (Thermo Fisher, A14527) in Expi293 expression medium (Thermo Fisher, A1435101) according to manufacturer instructions. The absence of mycoplasma contamination was verified using a Universal Mycoplasma Detection kit (ATCC, 30-1012K), with testing performed monthly. The sequences encoding full-length JUNV GPC MC2 strain (GenBank ID: BAA00964.2) (residues 1–485) and Machupo GPC Carvallo strain (GenBank ID: AAN05425.1) (residues 1–496) were cloned into a pVRC vector containing a C-terminal TEV cleavage site flanked by short linkers (GGSENLYFQGASGG) followed by a Twin-Strep tag (TS; WSHPQFEKGGGSGGGSGGGSWSHPQFEK), generating JUNV GPC MC2-TS and MACV GPC Carvallo-TS constructs. To generate the JUNV and MACV GPC-K33A-TS constructs for structural analysis, K33 was mutated to alanine by site-directed mutagenesis. For the SKI-1/S1P expression plasmid, the full-length gene was synthesized by Twist Bioscience (residues 1–1,052; GenBank ID: BAA07653.2) and cloned into a pVRC vector using Gibson assembly.

### Monoclonal antibodies and TfR1-sAD-Fc

Plasmids encoding monoclonal antibodies CR1-07, CR1-10 and CR1-28 in the pVRC8400 expression plasmid were previously described^20^, except that hexa-histidine tags at the C termini of the heavy chains were not included. A plasmid encoding anti-SARS-CoV antibody S309, used as an isotype control, in the pVRC8400 expression plasmid was previously described^58^. Plasmids encoding AHF1-B7, AHF3-C5, AHF3-E8.2, AHF2-A2, AHF4-F2 and AHF4-H10.2 were also in the pVRC8400 expression plasmid and generated as part of a separate study that used single B-cell sorting to isolate GP1-reactive monoclonal antibodies from the blood of AHF survivors. We generated a construct encoding TfR1-sAD-Fc based on the sequence for this construct available through the X-ray crystal structure of the soluble ectodomain of *N. albigula* TfR1 bound to MACV GP1 (PDB ID: 6S9J)^21^. The TfR1-sAD sequence was followed by a GGSGGS linker, followed by the constant region of human IgG1 cloned into the pVRC8400 expression vector. All pVRC8400 expression vectors used a tissue plasminogen activator (tPA) signal peptide for secreted expression.

Monoclonal antibodies and TfR1-sAD-Fc were expressed in Expi293F cells and transfected using the ExpiFectamine 293 transfection kit (Thermo Fisher, A14525) according to manufacturer instructions. On day 5 post transfection, cell culture supernatants were clarified by centrifugation at 4,000 × *g* for 20 min and passed through a 0.22-µm filter (VWR, 431118). Filtered supernatants were incubated with MabSelect PrismA resin (Cytiva, 17549801) overnight at 4 °C and subsequently washed with five column volumes of phosphate buffered saline (PBS). Proteins were eluted in 0.2 M glycine (pH 3.0) and neutralized with 1 M Tris (pH 9.0), before concentration in a 50-kDa Amicon centrifugal filter unit (Sigma-Aldrich, UFC905096). Proteins were further purified by size-exclusion chromatography on an ÄKTA Pure 25 purification system (Cytiva) with UNICORN v.7.8 using a Superdex 200 Increase 10/300 GL column (Cytiva) in PBS. All proteins eluted as single peaks. Antibody KL-AV-2A1 is a previously described antibody purified from the supernatant of a hybridoma^30^.

### Cell surface immunostaining experiments

Cell surface immunostaining experiments were carried out with JUNV MC2 GPC-TS or MACV Carvallo GPC-TS in the pVRC vector as described above. JUNV MC2 GPC-TS constructs containing substitutions E10A_SSP_, K33A_SSP_, H67A_GP1_, H128A_GP1_, Y157A_GP1_ or F427I_GP2_ were generated using site-directed mutagenesis. MACV Carvallo GPC-TS constructs containing the E10A_SSP_, K33A_SSP,_ H67A _GP1_ and F438I_GP2_ substitutions were also generated using site-directed mutagenesis. For cell surface immunostaining experiments, on day 0, HEK 293T cells were seeded into a T150 cell culture flask (Corning, 355001*)*. On day 1, when cells were 70–80% confluent, they were transfected using Lipofectamine 3000 (Thermo Fisher, L3000150) according to manufacturer instructions. After a 6-h incubation, the media were replaced with pre-warmed DMEM containing 10% (v/v) FBS (Atlas Biologicals, F-0500-D). On day 2, cells were collected using TrypsinLE (Thermo Fisher, 12604013) and passed through a cell strainer.

Following collection, cells were transferred to a V-bottom 96-well plate (Genesee Scientific, 91-419V) and spun at 500 × *g* for 4 min. Cell pellets were washed two times with PBS supplemented with 1% (w/v) bovine serum albumin (BSA; Sigma-Aldrich, A7906-500G) before the addition of monoclonal antibodies, TfR1-sAD-Fc, or an isotype control antibody (S309). A series of eight 10-fold dilutions starting at 100 μg ml^−1^ was added to cells with gentle mixing. After a 1 h incubation step at 4 °C, cells were spun again at 500 × g for 4 min and washed three times with PBS with 1% (w/v) BSA. AffiniPure F(ab’)_2_ goat anti-human Fc conjugated to *R*-phycoerythrin (Jackson ImmunoResearch, 109-116-098) (for human antibodies) diluted 1:200 in PBS + 1% (w/v) BSA was added to each well and allowed to incubate for 1 h at 4 °C. Following this incubation, cells were washed three times with PBS with 1% (w/v) BSA and once with PBS. Cells were then fixed with 2% (v/v) paraformaldehyde in PBS and analysed on an iQue3 Screener PLUS (IntelliCyt) with IntelliCyt ForeCyt Standard Edition version 8.1.7524 (Sartorius) software.

For assessing expression, on day 0, HEK 293T cells were seeded into a 12-well plate (Corning, 3513*)*. On day 1, when cells were 70–80% confluent, they were transfected using Lipofectamine 3000 (Thermo Fisher, L3000150) according to manufacturer instructions. After a 6 h incubation, the media were replaced with pre-warmed DMEM containing 10% (v/v) FBS (Atlas Biologicals, F-0500-D). On day 2, cells were collected using TrypsinLE (ThermoFisher, 12604013) and passed through a cell strainer.

Following collect, cells were transferred to a V-bottom 96-well plate (Genesee Scientific, 91-419 V) and spun at 500 × *g* for 4 min. Cell pellets were washed two times with PBS supplemented with 1% (w/v) BSA (Sigma-Aldrich, A7906-500G) before the addition of monoclonal antibodies, TfR1-sAD-Fc or an isotype control antibody. A single concentration of 20 μg ml^−1^ was added to cells with gentle mixing. After a 1-h incubation step at 4 °C, cells were spun down again at 500 × *g* for 4 min and washed three times with PBS with 1% (w/v) BSA. Either AffiniPure F(ab’)_2_ donkey anti-mouse Fc conjugated to *R*-phycoerythrin (Jackson ImmunoResearch, 715-116-0150) (for the murine antibody KL-AV-2A1) or AffiniPure F(ab’)_2_ goat anti-human Fc conjugated to *R*-phycoerythrin (Jackson ImmunoResearch, 109-116-098) diluted 1:200 in PBS + 1% (w/v) BSA was added to each well and allowed to incubate for 1 h at 4 °C. Following this incubation, cells were washed three times with PBS with 1% (w/v) BSA and once with PBS. Cells were then fixed with 2% (v/v) paraformaldehyde in PBS and analysed on an iQue3 Screener PLUS (IntelliCyt) with IntelliCyt ForeCyt Standard Edition v.8.1.7524 (Sartorius) software. Figure panels were generated in GraphPad Prism (v.10.1.2).

### Expression and purification of K33A_SSP_ JUNV and MACV GPC

Full-length JUNV (MC2 strain WT or with K33A_SSP_ substitution containing a GP2 C-terminal TS tag) and MACV GPC (Carvallo strain with the K33A_SSP_ substitution containing a GP2 C-terminal TS tag) for structural analysis were produced by transiently transfecting Expi293F cells (Thermo Fisher, A14527) using PEI MAX (Polysciences, 24765-100). Two days post transfection, cells were collected by centrifugation at 500 × *g* for 8 min. Cell pellets were then homogenized in lysis buffer (10 mM Tris, 200 mM NaCl, 100 µM MgCl_2_, 15% (v/v) glycerol, 1× protease inhibitor cocktail) (Apex Bio, K1007) for 1 h at 4 °C. Lysates were then spun at 28,000 × *g* for 25 min and the supernatants were discarded. Cell pellets were then resuspended in solubilization buffer (25 mM Tris, 200 mM NaCl, 500 µM ZnSO_4_, 15% (v/v) glycerol, 1% (w/v) *n*-dodecyl-β-d-maltopyranoside (DDM) (Anatrace, D310-25 GM), 0.1% (w/v) cholesteryl hemisuccinate (CHS; Anatrace, CH210 5 GM), 1× protease inhibitor cocktail (Apex Bio, K1007)) and allowed to solubilize for 4 h at 4 °C before ultracentrifugation at 265,000 × *g* for 30 min. Supernatants from this step were then incubated with StrepTactin XT resin (Cytiva, 29401324) overnight at 4 °C. The resins were washed with Tris-buffered saline (TBS) (25 mM Tris, 200 mM NaCl, pH 8) containing decreasing amounts of glycerol to 0.3% (v/v) while substituting DDM and CHS for increasing amounts of lauryl maltose neopentyl glycol (LMNG; Anatrace, NG310) until 0.03% (w/v) LMNG was reached. Proteins were eluted by incubating the resin with elution buffer (25 mM Tris, 200 mM NaCl, 500 µM ZnSO_4_, 0.3% (v/v) glycerol, 0.03% LMNG (w/v), 50 mM biotin) for 1 h.

### AlphaFold 3 modelling

Predicted structures for JUNV GPC MC2 strain (GenBank: BAA00964.2) and MACV GPC Carvallo strain (GenBank: AAN05425.1) were generated using AF3 (ref. ^45^). For JUNV GPC, modelling was performed with SSP (residues 2–58) containing the K33A_SSP_ substitution, GP1 (residues 59–251) and GP2 (residues 252–485), provided as separate entities each with three copies. For MACV GPC, modelling was also performed with SSP (residues 2–58) containing the K33A_SSP_ substitution, GP1 (residues 59–262) and GP2 (residues 263–496), provided as separate entities each with three copies. *N*-linked glycans were included at the predicted positions as post-translational modifications. Six zinc ions were also added as part of the predictions.

### JUNV GPC cryo-EM sample preparation and data processing

Purified JUNV GPC (WT or K33A_SSP_ mutant) used for sample vitrification was in buffer containing 25 mM Tris-HCl, 200 mM NaCl, 500 µM ZnSO_4_, 0.3% (v/v) glycerol, 0.03% LMNG (w/v) and 50 mM biotin (elution from Strep-Tactin XT column). A volume of 3.5 µl was deposited on glow-discharged (12 mA, 10 s; Pelco easiGlow, Ted Pella) graphene oxide lacey carbon copper grids, R1.2/1.3 (Electron Microscopy Sciences, GOLC300Cu50). Samples were vitrified using a Vitrobot Mark IV system (Thermo Fisher/FEI) with 6 s blotting time, 0 blot force, at 4 °C and 100% humidity. Micrographs were collected on a Titan Krios 300 kV microscope (Thermo Fisher) equipped with a Falcon4i direct electron detector using a defocus range of −0.8 to −2.1 μm. Automated single-particle data acquisition was performed with EPU (v.3.7), with a nominal magnification of ×165,000, which yielded a calibrated pixel size of 0.83 Å. Raw movies were processed in cryoSPARC (v.4.4.1)^59^, including motion correction and contrast transfer function (CTF) estimation. A total of 298,119 particles were picked using Topaz (v.0.2.5a) from 23,060 micrographs. Particles were extracted from micrographs with a box size of 480 pixels, and 2D classification was performed to discard bad particles. A total of 90,122 particles were converted to Relion format using csparc2star and used in 3D classification. One class containing 92% of the total particle stack was used in a subsequent auto-refine job, resulting in a resolution of 3.0 Å at a gold-standard Fourier shell correlation (GSFSC) threshold of 0.143. To facilitate model building, we used post processing of maps with DeepEMhancer^60^. Local resolution estimates were generated using ResMap^61^.

### JUNV GPC model building and structure validation

As starting points for model building of JUNV GPC, we used a combination of the coordinates from the cryo-EM structure of LASV GP2 (PDB: 7PUY)^28^, the X-ray crystal structure of JUNV GP1 (PDB: 5NUZ)^24^, the NMR solution structure of the JUNV GP2 C-terminal ZBD (PDB: 2L0Z)^38^, and docked these into maps using UCSF ChimeraX (v.1.9)^62^. An AF3 (ref. ^45^) model of JUNV GPC was also generated to guide model building. Metrics scored the majority of the AF3 model as confident, except for domain termini and loops, which tended to be scored as low confidence (Extended Data Fig. 9a). There was a noticeable deviation in the spacing of TM helices that prevented rigid-body docking of the full complex into cryo-EM maps. Therefore, individual subunits were extracted and docked using UCSF ChimeraX. Following real-space refinement of the JUNV GP1 crystal structure in Coot (v.0.9.8.92)^63^, the GP1 N- and C-terminal segments were manually built using the AF3 GP1 subunit as a guide. We also used the AF3-predicted GP2 domain and the ZBD NMR structure (PDB: 2L0Z)^38^ to guide model building and refinement in Coot. In addition, individual sections with poor fitting were deleted and manually rebuilt using LASV GP2 coordinates (from PDB ID: 7PUY)^28^ as a guide. Finally, portions of the AF3-predicted SSP were docked, with high-quality maps allowing for unambiguous iterative model building. We performed iterative rounds of model building and real-space refinement in Coot and refined models in Phenix (v.1.21.1)^64^. Altogether, we observed interpretable density for SSP residues 2–57, GP1 residues 60–247, and GP2 residues 269–318 and 331–485. We observed density for almost all the expected *N*-linked glycans. GP1 residue N95 lacked additional density to suggest glycosylation, consistent with previous crystal structures of JUNV GP1 (refs. ^20,22–24^). Poor map quality for the SSP *N*-myristoylation group did not allow us to unambiguously build a model for the lipid modification, so it was not included in the deposited coordinates. The final model was validated using MolProbity^65^.

### MACV GPC cryo-EM sample preparation and data processing

Purified MACV GPC (K33A_SSP_ mutant) used for sample vitrification was in buffer containing 25 mM Tris-HCl, 200 mM NaCl, 500 µM ZnSO_4_, 0.3% (v/v) glycerol, 0.03% LMNG (w/v) and 50 mM biotin (elution from Strep-Tactin XT column). A volume of 3.5 µl was deposited on glow-discharged (12 mA, 10 s; Pelco easiGlow, Ted Pella) graphene oxide lacey carbon copper grids, R1.2/1.3 (Electron Microscopy Sciences, GOLC300Cu50). Samples were vitrified using a Vitrobot Mark IV system (Thermo Fisher) with 6 s blotting time, 0 blot force, at 4 °C with 100% humidity. Micrographs were collected on a Titan Krios 300 kV microscope (Thermo Fisher) equipped with a Falcon4i direct electron detector using a defocus range of −0.8 to −2.1 μm. Automated single-particle data acquisition was performed with EPU (v.3.7), with a nominal magnification of ×165,000, which yielded a calibrated pixel size of 0.73 Å. Raw movies were processed in cryoSPARC (v.4.4.1)^59^, including motion correction and contrast transfer function (CTF) estimation. A total of 384,130 particles were picked using Topaz (v.0.2.5a) from 26,638 micrographs. Chosen particles were extracted from micrographs with a box size of 480 pixels and processed through a bifurcated approach resulting in two final maps. For one map (Map 1), 2D classification was performed to discard bad particles. A total of 186,510 particles from good class averages were selected for ab initio model generation (six classes), and the 100,386 particles belonging to three classes were selected for 3D classification. A class of 31,197 particles was then used for non-uniform refinement with C3 symmetry, resulting in a resolution of 2.86 Å at a GSFSC threshold of 0.143.

For the second map (Map 2), 384,130 particles were converted to Relion format using csparc2star and further subjected to 3D classification. One class containing 20% of particles was selected for auto-refinement using C3 symmetry, resulting in a resolution of 3.2 Å at a GSFSC threshold of 0.143. To facilitate model building, both maps were processed using DeepEMhancer^60^. Both maps were deposited because they differed in quality in certain segments that are likely flexible including the GP1 loop 10, the C terminus of GP1 and the ZBDs at the C terminus of GP2. Local resolution estimates were generated using ResMap^61^.

### MACV GPC model building and structure validation

As starting points for model building of MACV GPC, we used a combination of coordinates from the JUNV GPC model, the X-ray crystal structures of MACV GP1 (PDB: 2WFO and 7QU1)^23,26^ and an AF3 (ref. ^45^) model of MACV GPC as a general guide for individual domains (Extended Data Fig. 9f). We used rigid-body docking of the GP1 crystal structure (PDB: 2WFO)^26^ into cryo-EM maps using UCSF ChimeraX and manually built the GP1 N- and C-terminal regions. For SSP and GP2, the JUNV GPC cryo-EM model was primarily used for building; docking of JUNV subunits was followed by iterative rounds of mutating sections to the MACV sequence and refining the model. Docking was performed using UCSF ChimeraX^66^ and iterative rounds of model building were done in Coot (v.0.9.8.92)^63^, followed by real-space refinement in Phenix (v.1.21.1)^64^. On the basis of interpretable density, we built a model that included SSP residues 2–57, GP1 residues 60–256 and GP2 residues 280–496. We observed density for four predicted *N*-linked glycans on MACV GP1 and four on GP2. Poor map quality for the SSP *N*-myristoylation group did not allow us to unambiguously build a model for the lipid modification, so it was not included in the deposited coordinates. The model was validated using MolProbity^65^.

### Generation of split-GFP cell lines

We packaged transduction vectors by co-transfecting HEK 293T cells with plasmid pQCXIP-GFP1-10 (Addgene, 68715; a gift from Yutaka Hata)^67^ or pQCXIP-BSR-GFP11 (Addgene, 68716; a gift from Yutaka Hata)^67^, along with vectors expressing murine leukaemia virus (MLV) gag/pol and vesicular stomatitis virus G protein using Lipofectamine 3000 for transfection. After 48 h, supernatants were collected and filtered through a 0.22-μm syringe filter (76479-044). We transduced HEK 293T cells in a 6-well plate (Corning, 3516). Two days later, we replaced media with DMEM selection media supplemented with 10% (v/v) FBS, 25 mM HEPES (Gibco, 15630080) and 1 μg ml^−1^ puromycin. Cells (293T-GFP1-10 and 293T-GFP-11) were passaged three times in selection media and then twice in DMEM containing 10% (v/v) FBS before freezing stocks for long-term storage.

### Cell–cell fusion assays

To measure the pH-dependent membrane fusion activity of GPC mutants, we performed cell–cell fusion assays in Nunclon Delta 96-well microwell plates (Thermo Scientific, 167008). On day 0, 293T-GFP1-10 and 293T-GFP11 cell lines were seeded at a 1:1 ratio in the 96-well plate at a density of 3 × 10^4^ cells per well. On day 1, cells were transfected with Lipofectamine 3000 using 0.1 μg of GPC plasmids per well. Media were replaced with DMEM containing 10% (v/v) FBS after 6 h. On day 2, cells were imaged using an Incucyte automated live-cell imager (Sartorius) before starting the assay to visually assess confluence. The media were replaced with low-pH pulse media (DMEM containing 5% (v/v) FBS, 10 mM HEPES and 10 mM PIPES) with corresponding pH adjustments and allowed to incubate for 20 min before replacing with neutral-pH DMEM containing 10% (v/v) FBS and 25 mM HEPES. Cells were then imaged using Incucyte at 8 h after media replacement. Images were collected using a ×20 objective. GFP-positive signal was measured with a threshold greater than 1.5 green calibrated units (GCU) above background, using a surface-fit background subtraction method. The cell body area in each image was obtained by analysing phase-contrast images using the Incucyte S3 software (v.2023B). To calculate the fraction of cell fusion events, at the time point of 8 h post infection, the area of GFP signal above background was divided by the total area covered by cells. Data were plotted in GraphPad Prism (v.10.1.2)

### In silico modelling and MD simulations

The cryo-EM structures of the A33_SSP_ JUNV GPC and A33_SSP_ MACV GPC were prepared before modelling and simulations. The module ‘Protein Preparation in Schrödinger Maestro’^68^ was applied to cap termini, repair residues, optimize H-bond assignments and run restrained minimizations using default settings. In silico modelling of WT K33_SSP_ JUNV GPC or WT K33_SSP_ MACV GPC was performed using the 3D Builder module in Schrödinger Maestro. Protein models after in silico mutations and membrane embedding underwent the same preparation procedure.

Membrane Builder^69^ in CHARMM-GUI^70^ was used to build a viral membrane system. The mixed lipid ratio (DPPC/POPC/DPPE/POPE/DPPS/POPS/PSM/Chol = 4:6:12:18:4:6:20:30) was used in both leaflets to represent a liquid-ordered viral membrane^32–34^. TM residues on SSP chains and GP2 chains were selected to guide the placement of the lipid bilayer. The Schrödinger Desmond MD engine^71^ was used to perform MD simulations. An orthorhombic water box was applied to prepare membrane-embedded protein systems with a minimum distance of 10 Å to the edges from the top and bottom of the protein. Water molecules were described using the SPC model. Na^+^ and Cl^−^ ions were placed to create a physiological ionic concentration (150 mM NaCl) and neutralize the total net charge. All simulations were performed using the OPLS4 force field^72^. The ensemble class of NPT was selected with the simulation temperature set to 300 K (Nose–Hoover chain) and the pressure set to 1.01325 bar (Martyna–Tobias–Klein). A set of default minimization steps pre-defined in the Desmond protocol was adopted to relax the MD system. Before the full simulation, the relaxation of the membrane was set to 100 ns with positional restraints on the protein (Extended Data Fig. 7). The simulation of the full system was set to 500 ns for each membrane-embedded protein system (cryo-EM A33_SSP_ JUNV GPC, cryo-EM A33_SSP_ MACV GPC, modelled WT K33_SSP_ JUNV GPC and modelled WT K33_SSP_ MACV GPC). Each protein system underwent three replicate MD runs. One frame was recorded every 200 ps during the sampling phase. Post-simulation analysis of the RMSF and root-mean-square deviation (RMSD) was performed using a Schrödinger simulation interaction diagram. RMSF and RMSD values from the Cα of each residue were used for plotting.

### Statistical analysis in the methods

Data were deemed statistically significant when *P* < 0.05. Data from immunostaining experiments and cell–cell fusion assays were analysed using one-way or two-way analysis of variance (ANOVA) with multiple comparisons correction in GraphPad Prism (v.10.1.2). For statistical analysis of MD RMSF data, the data analysis function in Microsoft Excel (v.16.62) was used to conduct the unpaired, two-tailed Student’s *t*-test to compare mean RMSF values from two groups. *P* values are indicated in each of the figure legends.

### Reporting summary

Further information on research design is available in the Nature Portfolio Reporting Summary linked to this article.

## Supplementary information

Supplementary Information Supplementary Tables 1 and 3–5.

Reporting Summary

Supplementary Table 2 List of RMSD and RMSF values for MD simulations.

## Source data

Source Data Fig. 1 Statistical source data.

Source Data Fig. 2 Statistical source data.

Source Data Fig. 3 Statistical source data.

Source Data Fig. 5 Statistical source data.

Source Data Fig. 6 Statistical source data.

Source Data Extended Data Fig. 1 Unprocessed gels.

Source Data Extended Data Fig.e 3 Statistical source data.

Source Data Extended Data Fig. 7 Statistical source data.

Source Data Extended Data Fig. 8 Statistical source data.

## Data Availability

Protein Data Bank (PDB) and Electron Microscopy Data Bank (EMDB) identification numbers for the cryo-EM structures and maps reported in this manuscript are available under PDB: 9MT6 and EMD-48601 for JUNV GPC or PDB: 9MT2, and EMD-48598 for MACV GPC. All data that support the findings of this study are available within the Article and its supplementary information. Source data are provided with this paper. Reagents generated in this study are available from the corresponding author upon request with a completed material transfer agreement.

## Code availability

This manuscript does not report any new code.

## References

[1] Sironi, M., Forni, D. & de la Torre, J. C. Mammarenavirus genetic diversity and its biological implications. *Curr. Top. Microbiol. Immunol.* **439**, 265–303 (2023). 36592249 10.1007/978-3-031-15640-3_8

[2] Sarute, N. & Ross, S. R. New World arenavirus biology. *Annu. Rev. Virol.* **4**, 141–158 (2017). 28645238 10.1146/annurev-virology-101416-042001 PMC7478856

[3] de Mello Malta, F. et al. Sabia virus-like mammarenavirus in patient with fatal hemorrhagic fever, Brazil, 2020. *Emerg. Infect. Dis.* **26**, 1332–1334 (2020). 32441627 10.3201/eid2606.200099 PMC7258484

[4] Burri, D. J., da, Palma, J. R., Kunz, S. & Pasquato, A. Envelope glycoprotein of arenaviruses. *Viruses* **4**, 2162–2181 (2012). 23202458 10.3390/v4102162 PMC3497046

[5] Lenz, O., ter Meulen, J., Klenk, H. D., Seidah, N. G. & Garten, W. The Lassa virus glycoprotein precursor GP-C is proteolytically processed by subtilase SKI-1/S1P. *Proc. Natl Acad. Sci. USA* **98**, 12701–12705 (2001). 11606739 10.1073/pnas.221447598 PMC60117

[6] Beyer, W. R., Popplau, D., Garten, W., von Laer, D. & Lenz, O. Endoproteolytic processing of the lymphocytic choriomeningitis virus glycoprotein by the subtilase SKI-1/S1P. *J. Virol.* **77**, 2866–2872 (2003). 12584310 10.1128/JVI.77.5.2866-2872.2003 PMC149737

[7] Kunz, S., Edelmann, K. H., de la Torre, J. C., Gorney, R. & Oldstone, M. B. Mechanisms for lymphocytic choriomeningitis virus glycoprotein cleavage, transport, and incorporation into virions. *Virology* **314**, 168–178 (2003). 14517070 10.1016/s0042-6822(03)00421-5

[8] Lenz, O., ter Meulen, J., Feldmann, H., Klenk, H. D. & Garten, W. Identification of a novel consensus sequence at the cleavage site of the Lassa virus glycoprotein. *J. Virol.* **74**, 11418–11421 (2000). 11070044 10.1128/jvi.74.23.11418-11421.2000 PMC113249

[9] Radoshitzky, S. R. et al. Transferrin receptor 1 is a cellular receptor for New World haemorrhagic fever arenaviruses. *Nature* **446**, 92–96 (2007). 17287727 10.1038/nature05539 PMC3197705

[10] Radoshitzky, S. R. et al. Receptor determinants of zoonotic transmission of New World hemorrhagic fever arenaviruses. *Proc. Natl Acad. Sci. USA* **105**, 2664–2669 (2008). 18268337 10.1073/pnas.0709254105 PMC2268193

[11] Helguera, G. et al. An antibody recognizing the apical domain of human transferrin receptor 1 efficiently inhibits the entry of all new world hemorrhagic fever arenaviruses. *J. Virol.* **86**, 4024–4028 (2012). 22278244 10.1128/JVI.06397-11 PMC3302512

[12] York, J., Romanowski, V., Lu, M. & Nunberg, J. H. The signal peptide of the Junin arenavirus envelope glycoprotein is myristoylated and forms an essential subunit of the mature G1-G2 complex. *J. Virol.* **78**, 10783–10792 (2004). 15367645 10.1128/JVI.78.19.10783-10792.2004 PMC516395

[13] York, J. & Nunberg, J. H. Role of the stable signal peptide of Junin arenavirus envelope glycoprotein in pH-dependent membrane fusion. *J. Virol.* **80**, 7775–7780 (2006). 16840359 10.1128/JVI.00642-06 PMC1563716

[14] York, J. & Nunberg, J. H. Intersubunit interactions modulate pH-induced activation of membrane fusion by the Junin virus envelope glycoprotein GPC. *J. Virol.* **83**, 4121–4126 (2009). 19224989 10.1128/JVI.02410-08 PMC2668491

[15] Enria, D. A. & Barrera Oro, J. G. Junin virus vaccines. *Curr. Top. Microbiol. Immunol.* **263**, 239–261 (2002). 11987817 10.1007/978-3-642-56055-2_12

[16] Albarino, C. G. et al. The major determinant of attenuation in mice of the Candid1 vaccine for Argentine hemorrhagic fever is located in the G2 glycoprotein transmembrane domain. *J. Virol.* **85**, 10404–10408 (2011). 21795336 10.1128/JVI.00856-11 PMC3196416

[17] Seregin, A. V. et al. The glycoprotein precursor gene of Junin virus determines the virulence of the Romero strain and the attenuation of the Candid #1 strain in a representative animal model of Argentine hemorrhagic fever. *J. Virol.* **89**, 5949–5956 (2015). 25810546 10.1128/JVI.00104-15 PMC4442433

[18] Patterson, M. et al. A substitution in the transmembrane region of the glycoprotein leads to an unstable attenuation of Machupo virus. *J. Virol.* **88**, 10995–10999 (2014). 25031335 10.1128/JVI.01007-14 PMC4178861

[19] York, J., Dai, D., Amberg, S. M. & Nunberg, J. H. pH-induced activation of arenavirus membrane fusion is antagonized by small-molecule inhibitors. *J. Virol.* **82**, 10932–10939 (2008). 18768973 10.1128/JVI.01140-08 PMC2573205

[20] Clark, L. E. et al. Vaccine-elicited receptor-binding site antibodies neutralize two New World hemorrhagic fever arenaviruses. *Nat. Commun.* **9**, 1884 (2018). 29760382 10.1038/s41467-018-04271-z PMC5951886

[21] Cohen-Dvashi, H. et al. Rational design of universal immunotherapy for TfR1-tropic arenaviruses. *Nat. Commun.* **11**, 67 (2020). 31900422 10.1038/s41467-019-13924-6 PMC6941993

[22] Mahmutovic, S. et al. Molecular basis for antibody-mediated neutralization of New World hemorrhagic fever mammarenaviruses. *Cell Host Microbe* **18**, 705–713 (2015). 26651946 10.1016/j.chom.2015.11.005 PMC4685251

[23] Ng, W. M. et al. Contrasting modes of New World arenavirus neutralization by immunization-elicited monoclonal antibodies. *mBio* **13**, e0265021 (2022). 35315691 10.1128/mbio.02650-21 PMC9040744

[24] Zeltina, A. et al. Convergent immunological solutions to Argentine hemorrhagic fever virus neutralization. *Proc. Natl Acad. Sci. USA* **114**, 7031–7036 (2017). 28630325 10.1073/pnas.1702127114 PMC5502616

[25] York, J. & Nunberg, J. H. Myristoylation of the arenavirus envelope glycoprotein stable signal peptide is critical for membrane fusion but dispensable for virion morphogenesis. *J. Virol.* **90**, 8341–8350 (2016). 27412594 10.1128/JVI.01124-16 PMC5008094

[26] Bowden, T. A. et al. Unusual molecular architecture of the machupo virus attachment glycoprotein. *J. Virol.* **83**, 8259–8265 (2009). 19494008 10.1128/JVI.00761-09 PMC2715760

[27] Abraham, J., Corbett, K. D., Farzan, M., Choe, H. & Harrison, S. C. Structural basis for receptor recognition by New World hemorrhagic fever arenaviruses. *Nat. Struct. Mol. Biol.* **17**, 438–444 (2010). 20208545 10.1038/nsmb.1772 PMC2920743

[28] Katz, M. et al. Structure and receptor recognition by the Lassa virus spike complex. *Nature* **603**, 174–179 (2022). 35173332 10.1038/s41586-022-04429-2

[29] Eilon-Ashkenazy, M. et al. The structure of the Lujo virus spike complex. *Nat. Commun.* **15**, 7175 (2024). 39169025 10.1038/s41467-024-51606-0 PMC11339409

[30] Amanat, F. et al. Monoclonal antibodies with neutralizing activity and Fc-effector functions against the Machupo virus glycoprotein. *J. Virol*. **94**, e01741-19 (2020). 10.1128/JVI.01741-19 PMC7022345 31801871

[31] Droniou-Bonzom, M. E. et al. Substitutions in the glycoprotein (GP) of the Candid#1 vaccine strain of Junin virus increase dependence on human transferrin receptor 1 for entry and destabilize the metastable conformation of GP. *J. Virol.* **85**, 13457–13462 (2011). 21976641 10.1128/JVI.05616-11 PMC3233171

[32] Ivanova, P. T. et al. Lipid composition of viral envelope of three strains of influenza virus – not all viruses are created equal. *ACS Infect. Dis.* **1**, 399–452 (2015). 26448476 10.1021/acsinfecdis.5b00040 PMC4593503

[33] Brugger, B. et al. The HIV lipidome: a raft with an unusual composition. *Proc. Natl Acad. Sci. USA* **103**, 2641–2646 (2006). 16481622 10.1073/pnas.0511136103 PMC1413831

[34] Woo, H. et al. Developing a fully glycosylated full-length SARS-CoV-2 spike protein model in a viral membrane. *J. Phys. Chem. B* **124**, 7128–7137 (2020). 32559081 10.1021/acs.jpcb.0c04553 PMC7341691

[35] York, J. & Nunberg, J. H. Epistastic interactions within the Junin virus envelope glycoprotein complex provide an evolutionary barrier to reversion in the live-attenuated Candid#1 vaccine. *J. Virol.* **92**, e01682-17 (2018). 10.1128/JVI.01682-17 PMC5730776 29070682

[36] Eichler, R. et al. Lassa virus glycoprotein signal peptide displays a novel topology with an extended endoplasmic reticulum luminal region. *J. Biol. Chem.* **279**, 12293–12299 (2004). 14709548 10.1074/jbc.M312975200

[37] Agnihothram, S. S., York, J., Trahey, M. & Nunberg, J. H. Bitopic membrane topology of the stable signal peptide in the tripartite Junin virus GP-C envelope glycoprotein complex. *J. Virol.* **81**, 4331–4337 (2007). 17267481 10.1128/JVI.02779-06 PMC1866146

[38] Briknarova, K., Thomas, C. J., York, J. & Nunberg, J. H. Structure of a zinc-binding domain in the Junin virus envelope glycoprotein. *J. Biol. Chem.* **286**, 1528–1536 (2011). 21068387 10.1074/jbc.M110.166025 PMC3020761

[39] York, J. & Nunberg, J. H. A novel zinc-binding domain is essential for formation of the functional Junin virus envelope glycoprotein complex. *J. Virol.* **81**, 13385–13391 (2007). 17928348 10.1128/JVI.01785-07 PMC2168868

[40] Bederka, L. H., Bonhomme, C. J., Ling, E. L. & Buchmeier, M. J. Arenavirus stable signal peptide is the keystone subunit for glycoprotein complex organization. *mBio* **5**, e02063 (2014). 25352624 10.1128/mBio.02063-14 PMC4217180

[41] Hastie, K. M. et al. Structural basis for antibody-mediated neutralization of Lassa virus. *Science* **356**, 923–928 (2017). 28572385 10.1126/science.aam7260 PMC6007842

[42] Buck, T. K. et al. Neutralizing antibodies against Lassa virus lineage I. *mBio* **13**, e0127822 (2022). 35730904 10.1128/mbio.01278-22 PMC9426445

[43] Moon-Walker, A. et al. Structural basis for antibody-mediated neutralization of lymphocytic choriomeningitis virus. *Cell Chem. Biol.* **30**, 403–411.e4 (2023). 36990092 10.1016/j.chembiol.2023.03.005 PMC11090681

[44] Hastie, K. M. et al. Crystal structure of the prefusion surface glycoprotein of the prototypic arenavirus LCMV. *Nat. Struct. Mol. Biol.* **23**, 513–521 (2016). 27111888 10.1038/nsmb.3210 PMC4945123

[45] Abramson, J. et al. Accurate structure prediction of biomolecular interactions with AlphaFold 3. *Nature* **630**, 493–500 (2024). 38718835 10.1038/s41586-024-07487-w PMC11168924

[46] Shankar, S. et al. Small-molecule fusion inhibitors bind the pH-sensing stable signal peptide-GP2 subunit interface of the Lassa virus envelope glycoprotein. *J. Virol.* **90**, 6799–6807 (2016). 27194767 10.1128/JVI.00597-16 PMC4944282

[47] Bolken, T. C. et al. Identification and characterization of potent small molecule inhibitor of hemorrhagic fever New World arenaviruses. *Antivir. Res.* **69**, 86–97 (2006). 16343651 10.1016/j.antiviral.2005.10.008 PMC7114356

[48] Larson, R. A. et al. Identification of a broad-spectrum arenavirus entry inhibitor. *J. Virol.* **82**, 10768–10775 (2008). 18715909 10.1128/JVI.00941-08 PMC2573164

[49] Burgeson, J. R. et al. Lead optimization of an acylhydrazone scaffold possessing antiviral activity against Lassa virus. *Bioorg. Med. Chem. Lett.* **23**, 5840–5843 (2013). 24064500 10.1016/j.bmcl.2013.08.103 PMC3836667

[50] Thomas, C. J. et al. A specific interaction of small molecule entry inhibitors with the envelope glycoprotein complex of the Junin hemorrhagic fever arenavirus. *J. Biol. Chem.* **286**, 6192–6200 (2011). 21159779 10.1074/jbc.M110.196428 PMC3057843

[51] Lee, A. M. et al. Unique small molecule entry inhibitors of hemorrhagic fever arenaviruses. *J. Biol. Chem.* **283**, 18734–18742 (2008). 18474596 10.1074/jbc.M802089200 PMC2441566

[52] Whitby, L. R., Lee, A. M., Kunz, S., Oldstone, M. B. & Boger, D. L. Characterization of lassa virus cell entry inhibitors: determination of the active enantiomer by asymmetric synthesis. *Bioorg. Med. Chem. Lett.* **19**, 3771–3774 (2009). 19428249 10.1016/j.bmcl.2009.04.098 PMC2742202

[53] Thomas, C. J. et al. Biochemical reconstitution of hemorrhagic-fever arenavirus envelope glycoprotein-mediated membrane fusion. *PLoS ONE* **7**, e51114 (2012). 23226473 10.1371/journal.pone.0051114 PMC3511403

[54] Messina, E. L., York, J. & Nunberg, J. H. Dissection of the role of the stable signal peptide of the arenavirus envelope glycoprotein in membrane fusion. *J. Virol.* **86**, 6138–6145 (2012). 22438561 10.1128/JVI.07241-11 PMC3372177

[55] Li, H. et al. A cocktail of protective antibodies subverts the dense glycan shield of Lassa virus. *Sci. Transl. Med.* **14**, eabq0991 (2022). 36288283 10.1126/scitranslmed.abq0991 PMC10084740

[56] Gorman, J. et al. Cleavage-intermediate Lassa virus trimer elicits neutralizing responses, identifies neutralizing nanobodies, and reveals an apex-situated site-of-vulnerability. *Nat. Commun.* **15**, 285 (2024). 38177144 10.1038/s41467-023-44534-y PMC10767048

[57] Perrett, H. R. et al. Structural conservation of Lassa virus glycoproteins and recognition by neutralizing antibodies. *Cell Rep.* **42**, 112524 (2023). 37209096 10.1016/j.celrep.2023.112524 PMC10242449

[58] Nabel, K. G. et al. Structural basis for continued antibody evasion by the SARS-CoV-2 receptor binding domain. *Science* **375**, eabl6251 (2022). 34855508 10.1126/science.abl6251 PMC9127715

[59] Punjani, A., Rubinstein, J. L., Fleet, D. J. & Brubaker, M. A. cryoSPARC: algorithms for rapid unsupervised cryo-EM structure determination. *Nat. Methods* **14**, 290–296 (2017). 28165473 10.1038/nmeth.4169

[60] Sanchez-Garcia, R. et al. DeepEMhancer: a deep learning solution for cryo-EM volume post-processing. *Commun. Biol.* **4**, 874 (2021). 34267316 10.1038/s42003-021-02399-1 PMC8282847

[61] Kucukelbir, A., Sigworth, F. J. & Tagare, H. D. Quantifying the local resolution of cryo-EM density maps. *Nat. Methods* **11**, 63–65 (2014). 24213166 10.1038/nmeth.2727 PMC3903095

[62] Pettersen, E. F. et al. UCSF Chimera—a visualization system for exploratory research and analysis. *J. Comput. Chem.* **25**, 1605–1612 (2004). 15264254 10.1002/jcc.20084

[63] Emsley, P., Lohkamp, B., Scott, W. G. & Cowtan, K. Features and development of Coot. *Acta Crystallogr. D* **66**, 486–501 (2010). 20383002 10.1107/S0907444910007493 PMC2852313

[64] Adams, P. D. et al. The Phenix software for automated determination of macromolecular structures. *Methods* **55**, 94–106 (2011). 21821126 10.1016/j.ymeth.2011.07.005 PMC3193589

[65] Williams, C. J. et al. MolProbity: more and better reference data for improved all-atom structure validation. *Protein Sci.* **27**, 293–315 (2018). 29067766 10.1002/pro.3330 PMC5734394

[66] Goddard, T. D. et al. UCSF ChimeraX: meeting modern challenges in visualization and analysis. *Protein Sci.* **27**, 14–25 (2018). 28710774 10.1002/pro.3235 PMC5734306

[67] Kodaka, M. et al. A new cell-based assay to evaluate myogenesis in mouse myoblast C2C12 cells. *Exp. Cell. Res.* **336**, 171–181 (2015). 26116467 10.1016/j.yexcr.2015.06.015

[68] Sastry, G. M., Adzhigirey, M., Day, T., Annabhimoju, R. & Sherman, W. Protein and ligand preparation: parameters, protocols, and influence on virtual screening enrichments. *J. Comput. Aided Mol. Des.* **27**, 221–234 (2013). 23579614 10.1007/s10822-013-9644-8

[69] Lee, J. et al. CHARMM-GUI membrane builder for complex biological membrane simulations with glycolipids and lipoglycans. *J. Chem. Theory Comput.* **15**, 775–786 (2019). 30525595 10.1021/acs.jctc.8b01066

[70] Jo, S. et al. CHARMM-GUI 10 years for biomolecular modeling and simulation. *J. Comput. Chem.* **38**, 1114–1124 (2017). 27862047 10.1002/jcc.24660 PMC5403596

[71] Bowers, K. J. et al. Scalable algorithms for molecular dynamics simulations on commodity clusters. In *Proc. 2006 ACM/IEEE Conference on Supercomputing* 84–es (ACM, 2006).

[72] Lu, C. et al. OPLS4: improving force field accuracy on challenging regimes of chemical space. *J. Chem. Theory Comput.* **17**, 4291–4300 (2021). 34096718 10.1021/acs.jctc.1c00302

[73] Robert, X. & Gouet, P. Deciphering key features in protein structures with the new ENDscript server. *Nucleic Acids Res.* **42**, W320–W324 (2014). 24753421 10.1093/nar/gku316 PMC4086106

