# Supplementary material for: Molecular organization of the New World arenavirus spike glycoprotein complex
Source: Nat Microbiol. 2025 Aug 8;10(9):2207–20. doi: 10.1038/s41564-025-02085-6 (PMC12408356; doi:10.1038/s41564-025-02085-6)
Supplement: Supplementary file 1 — Supplementary Tables 1 and 3–5. [file 41564_2025_2085_MOESM1_ESM.pdf]

nature microbiology

Supplementary information

https://doi.org/10.1038/s41564-025-02085-6

Molecular organization of the New World arenavirus spike glycoprotein complex

In the format provided by the authors and unedited

Supplementary Table 1. Cryo-EM data collection and validation statistics.

	JUNV GPC	MACV GPC (Map 1)	MACV GPC (Map 2)
Microscope	Titan Krios	Titan Krios	
Detector	Falcon4i	Falcon4i	
Magnification	165,000x	165,000x	
Voltage (kV)	300	300	
Pixel Size (Å)	0.83*	0.73*	
Electron dose (e/Å^2)	50.0	51.4	
Defocus range (μm)	-0.8 to -2.1	-0.8 to -2.1	
Micrographs	23,060	26,638	
Final particles	83,055	31,197	77,380
Symmetry imposed	C3	C3	
FSC threshold	0.143	0.143	0.143
Map resolution (Å)	3.0	2.9	3.2
**Model refinement and validation**			
Initial model used	7PUY, 5NUZ, 2L0Z, AlphaFold 3	2WFO, 7QU1, 2L0Z, AlphaFold 3	
R.m.s deviations			
Bonds lengths (Å)	0.004	0.004	
Bonds angles (º)	0.685	0.915	
Validation			
Molprobity score	1.64	1.75	
Clashscore	7	9	
Ramachandran plot			
Favored (%)	96.2	96.8	
Allowed (%)	3.8	3.2	
Disallowed (%)	0	0	

Supplementary Table 3. Summary of GPC lipid pocket occupancies from MD simulations.

Membrane embedded protein systems	Replicate 1			Replicate 2			Replicate 3		
	SSP_A	SSP_B	SSP_C	SSP_A	SSP_B	SSP_C	SSP_A	SSP_B	SSP_C
**JUNV GPC A33$_{SSP}$**	POPC	PSM	POPC	POPC	PSM	POPE/ POPS[1]	POPC	POPS	POPE
**JUNV GPC K33$_{SSP}$ (*in silico*)**	POPC	POPS	POPS	POPC	Empty	K33	DPPE	POPC	K33
**MACV GPC A33$_{SSP}$**	POPE	POPC	PSM	POPE	POPC	Empty	POPE	POPC	POPC/ PSM[2]
**MACV GPC K33$_{SSP}$ (*in silico*)**	POPE	K33	K33	POPS	CHL	K33	K33	POPE	PSM

[1] POPE from the upper leaflet and POPS from the lower leaflet approach the pocket simultaneously.
[2] POPC from the upper leaflet and PSM from the lower leaflet approach the pocket simultaneously.

Supplementary Table 4. Summary of JUNV and MACV mutational analyses.

Virus	Subunit	Substitution	Effect based on structures	Effect observed in assays
**JUNV GPC**	SSP	E10A	Disrupt GP1/GP2/SSP interaction	Fusion at pH > 5.5*
		K33A	Improved hydrophobic packing in TM region	No fusion at any pH
	GP1	H67A	Disrupt GP1/SSP interaction	Increase fusion at pH 5.5*
		H128A	Disrupt GP1 apex trimerization contact	No effect
		Y157A	Disrupt GP1 apex trimerization contact	Increase cell-cell fusion at pH 5.5
	GP2	F427I	Weakens hydrophobic packing in TM region	Fusion at pH > 5.5*
**MACV GPC**	SSP	E10A	Disrupt GP1/GP2/SSP interaction	Very weak fusion at pH > 5.5*
		K33A	Improved hydrophobic packing in TM region	No fusion at any pH
	GP1	H67A	Disrupt GP1/SSP interaction	No effect
	GP2	F438I	Weaken hydrophobic packing in TM region	Fusion at pH > 5.5*

***** Denotes substitutions that likely result in increased GP1 shedding based on differences in TfR1-sAD-Fc and KL-AV-2A1 staining.

Supplementary Table 5. Summary of JUNV GPC mutants resistant to small-molecule inhibitors.

Subunit	Substitution	Inhibitor	Reference
**SSP**	P12A	ST-193	Messina et al. (2012)
	T13A	ST-193	Messina et al. (2012)
	K33H/R	ST-193	York et al. (2008)
**GP2**	I347A	ST-294	York et al. (2008)
	D400A	ST-294	York et al. (2008)
	T418N	ST-294	York et al. (2008)
	L420T	ST-294	York et al. (2008)
	A435I	ST-294	York et al. (2008)
	F427A	ST-294	York et al. (2008)
	F438I	ST-294	York et al. (2008)
