# Supplementary material for: Molecular organization of the New World arenavirus spike glycoprotein complex
Source: Nat Microbiol. 2025 Aug 8;10(9):2207–20. doi: 10.1038/s41564-025-02085-6 (PMC12408356; doi:10.1038/s41564-025-02085-6)
Supplement: Supplementary file 2 — Reporting Summary [file 41564_2025_2085_MOESM2_ESM.pdf]

nature portfolio

Corresponding author(s): Jonathan Abraham

Last updated by author(s): Jul 8, 2025

Reporting Summary

Nature Portfolio wishes to improve the reproducibility of the work that we publish. This form provides structure for consistency and transparency in reporting. For further information on Nature Portfolio policies, see our Editorial Policies and the Editorial Policy Checklist.

Statistics

For all statistical analyses, confirm that the following items are present in the figure legend, table legend, main text, or Methods section.

n/a	Confirmed	
☐	☒	The exact sample size (*n*) for each experimental group/condition, given as a discrete number and unit of measurement
☐	☒	A statement on whether measurements were taken from distinct samples or whether the same sample was measured repeatedly
☐	☒	The statistical test(s) used AND whether they are one- or two-sided<br>*Only common tests should be described solely by name; describe more complex techniques in the Methods section.*
☐	☒	A description of all covariates tested
☐	☒	A description of any assumptions or corrections, such as tests of normality and adjustment for multiple comparisons
☐	☒	A full description of the statistical parameters including central tendency (e.g. means) or other basic estimates (e.g. regression coefficient) AND variation (e.g. standard deviation) or associated estimates of uncertainty (e.g. confidence intervals)
☐	☒	For null hypothesis testing, the test statistic (e.g. *F*, *t*, *r*) with confidence intervals, effect sizes, degrees of freedom and *P* value noted<br>*Give P values as exact values whenever suitable.*
☒	☐	For Bayesian analysis, information on the choice of priors and Markov chain Monte Carlo settings
☒	☐	For hierarchical and complex designs, identification of the appropriate level for tests and full reporting of outcomes
☒	☐	Estimates of effect sizes (e.g. Cohen's *d*, Pearson's *r*), indicating how they were calculated

Our web collection on statistics for biologists contains articles on many of the points above.

Software and code

Policy information about availability of computer code

Data collection	ForeCyt (Sartorius) (version 8.1.7524), EPU (version 2.13), Incucyte 53 Software (version 2023B).
Data analysis	FlowJo (version 10.6.2), GraphPad Prism (version 10.1.2), RELION (version 3.1.4), cryoSPARC (version 4.4.1), DeepEMhancer (version 20210511), UCSF ChimeraX (version 1.9), PyMOL (version 2.5.4), Incucyte 53 Software (version 2023B), ResMap (version 1.1.4), Coot (version 0.9.8.92), PHENIX (version 1.21-1).

For manuscripts utilizing custom algorithms or software that are central to the research but not yet described in published literature, software must be made available to editors and reviewers. We strongly encourage code deposition in a community repository (e.g. GitHub). See the Nature Portfolio guidelines for submitting code & software for further information.

Data

Policy information about availability of data

All manuscripts must include a data availability statement. This statement should provide the following information, where applicable:

- Accession codes, unique identifiers, or web links for publicly available datasets
- A description of any restrictions on data availability
- For clinical datasets or third party data, please ensure that the statement adheres to our policy

Protein Data Bank (PDB) and Electron Microscopy Data Bank (EMDB) identification numbers for the cryo-EM structures and maps reported in this manuscript are

available under 9MT6 and EMD-48601 for JUNV GPC or 9MT2 and EMD-48598 for MACV GPC. All data that support the findings of this study are available within the article and its supplementary information. Source data are provided.

Research involving human participants, their data, or biological material

Policy information about studies with human participants or human data. See also policy information about sex, gender (identity/presentation), and sexual orientation and race, ethnicity and racism.

Reporting on sex and gender	*Use the terms sex (biological attribute) and gender (shaped by social and cultural circumstances) carefully in order to avoid confusing both terms. Indicate if findings apply to only one sex or gender; describe whether sex and gender were considered in study design; whether sex and/or gender was determined based on self-reporting or assigned and methods used. Provide in the source data disaggregated sex and gender data, where this information has been collected, and if consent has been obtained for sharing of individual-level data; provide overall numbers in this Reporting Summary. Please state if this information has not been collected. Report sex- and gender-based analyses where performed, justify reasons for lack of sex- and gender-based analysis.*
Reporting on race, ethnicity, or other socially relevant groupings	*Please specify the socially constructed or socially relevant categorization variable(s) used in your manuscript and explain why they were used. Please note that such variables should not be used as proxies for other socially constructed/relevant variables (for example, race or ethnicity should not be used as a proxy for socioeconomic status). Provide clear definitions of the relevant terms used, how they were provided (by the participants/respondents, the researchers, or third parties), and the method(s) used to classify people into the different categories (e.g. self-report, census or administrative data, social media data, etc.) Please provide details about how you controlled for confounding variables in your analyses.*
Population characteristics	*Describe the covariate-relevant population characteristics of the human research participants (e.g. age, genotypic information, past and current diagnosis and treatment categories). If you filled out the behavioural & social sciences study design questions and have nothing to add here, write "See above."*
Recruitment	*Describe how participants were recruited. Outline any potential self-selection bias or other biases that may be present and how these are likely to impact results.*
Ethics oversight	*Identify the organization(s) that approved the study protocol.*

Note that full information on the approval of the study protocol must also be provided in the manuscript.

Field-specific reporting

Please select the one below that is the best fit for your research. If you are not sure, read the appropriate sections before making your selection.

☒ Life sciences ☐ Behavioural & social sciences ☐ Ecological, evolutionary & environmental sciences

For a reference copy of the document with all sections, see nature.com/documents/nr-reporting-summary-flat.pdf

Life sciences study design

All studies must disclose on these points even when the disclosure is negative.

Sample size	No sample size calculations were performed to power each study and no statistical methods were used to predetermine sample size. Cell based experiments (binding experiments) were performed at least three times in triplicate, as noted in figure legends. The total numbers of particles picked during cryo-EM data analysis are provided in the Methods and Supplementary Information.
Data exclusions	No data from cell-based experiments were excluded. Particles without high-resolution features were discarded during data analysis as noted in the Extended Data This is standard practice in the field of cryo-EM.
Replication	Cell-based experiments were performed at least three times independently, with each experiment containing at least triplicates for every treatment. Binding assays (e.g., immunostaining) we performed at least three times with representative data shown. n values are defined and provided in each figure legend. Molecular dynamics simulations were performed three times independently. All attempts to replicate results were successful.
Randomization	For cell-based studies (e.g., immunostaining experiments), sample allocation was not randomized because the results are quantitative and did not require subjective measurement or interpretation. Please note that this practice is standard in the field.
Blinding	The investigators were not blinded to the allocation during experiments or to outcome assessment. Blinding was not deemed necessary because the results are quantitative and did not require subjective judgment or interpretation. Blinding is also not typically used in the field for similar studies.

Reporting for specific materials, systems and methods

We require information from authors about some types of materials, experimental systems and methods used in many studies. Here, indicate whether each material, system or method listed is relevant to your study. If you are not sure if a list item applies to your research, read the appropriate section before selecting a response.

Materials & experimental systems

n/a	Involved in the study
☐	☒ Antibodies
☐	☒ Eukaryotic cell lines
☒	☐ Palaeontology and archaeology
☒	☐ Animals and other organisms
☒	☐ Clinical data
☒	☐ Dual use research of concern
☒	☐ Plants

Methods

n/a	Involved in the study
☒	☐ ChIP-seq
☐	☒ Flow cytometry
☒	☐ MRI-based neuroimaging

Antibodies

Antibodies used

1. R phycoerythrin (PE) coupled goat anti human F(ab')2 fragment (Jackson ImmunoResearch Cat#: 109 116 098) 1:200 dilution
2. R phycoerythrin (PE) couples donkey anti mouse F(ab')2 fragment (Jackson ImmunoResearch Cat# 715-116-150) 1:200 dilution
3. Monoclonal antibodies CR1-07, CR1-10, CR1-28 (developed in previous study - doi.org/10.1038/s41467-018-04271-z). Used in 10-fold dilution series of eight, starting from 100 ug/ml.
4. Monoclonal antibodies AHF2-A2, AHF1-B7, AHF3-C5, AHF3-E8.2, AHF4-F2, AHF4-H10.2 (developed in a manuscript in preparation). Used in a 10-fold dilution series of eight, starting from 100 ug/ml. AHF3-E8.2 was also used at a concentration of 20 ug/ml for cell surface staining.
5. Monoclonal antibody KL-AV-2A1 (developed in a previous study - doi.org/10.1128/msphere.00189-18). Used at a concentration of 20 ug/ml for cell surface staining

Validation

l. R-phycoerythrin (PE)-coupled goat anti-human F(ab')2 fragment: Commercially validated. Specificity for Fc but not Fab of human IgG heavy chain was confirmed by immunoelectrophoresis and/or ELISA. Does not cross-react with human IgM or IgA, or against non-immunoglobulin serum proteins. ELISA and/or solid-phase adsorption were used to ensure minimal cross-reaction with bovine, horse and mouse serum proteins, but it may cross-react with immunoglobulins from other species.
2. R-phycoerythrin (PE)-coupled donkey anti mouse F(ab')2 fragment: Commercially validated Specificity for Fc but not Fab of mouse IgG heavy chain was confirmed by immunoelectrophoresis and/or ELISA. Does not cross-react with human IgM or IgA, or against non-immunoglobulin serum proteins. ELISA and/or solid-phase adsorption were used to ensure minimal cross-reaction with bovine, horse and mouse serum proteins, but it may cross-react with immunoglobulins from other species.
3. JUNV GP1 reactivity of monoclonal antibodies CR1-07, CR1-10, CR1-28 were determined by ELISA, surface plasmon resonance, and pseudovirus neutralization as part of previous study (doi.org/10.1038/s41467-018-04271-z). CR1-07 was also found to cross-react with MACV GP1.
4. JUNV GP1 reactivity of monoclonal antibodies AHF2-A2, AHF1-B7, AHF3-C5, AHF3-E8.2, AHF4-F2, AHF4-H10.2 was demonstrated by ELISA as part of a separate study. AHF3-E8.2 and AHF1-B7 were demonstrated to cross-react with MACV GP1 by biolayer interferometry. AHF2-A2, AHF4-F2, and AHF4-H10.2 were demonstrated to cross-react with MACV in pseudovirus neutralization assays.
5. JUNV and MACV GP2 cross-reactivity of KL-AV-2A1 was determined by ELISA and immunofluorescence staining in a previous study (doi.org/10.1128/msphere.00189-18)

Eukaryotic cell lines

Policy information about cell lines and Sex and Gender in Research

Cell line source(s)

HEK293T cells were obtained from ATCC (CRL-11268). Expi293F cells were obtained from ThermoFisher Scientific (Cat# A14527).

Authentication

Cell lines were obtained directly from commercial vendors but they were not authenticated once received.

Mycoplasma contamination

We confirmed the absence of mycoplasma in all immortalized cell lines through monthly testing using an e-Myco PCR detection kit.

Commonly misidentified lines
(See ICLAC register)

None.

Plants

Seed stocks

Report on the source of all seed stocks or other plant material used. If applicable, state the seed stock centre and catalogue number. If plant specimens were collected from the field, describe the collection location, date and sampling procedures.

Novel plant genotypes

Describe the methods by which all novel plant genotypes were produced. This includes those generated by transgenic approaches, gene editing, chemical/radiation-based mutagenesis and hybridization. For transgenic lines, describe the transformation method, the number of independent lines analyzed and the generation upon which experiments were performed. For gene-edited lines, describe the editor used, the endogenous sequence targeted for editing, the targeting guide RNA sequence (if applicable) and how the editor was applied.

Authentication

Describe any authentication procedures for each seed stock used or novel genotype generated. Describe any experiments used to assess the effect of a mutation and, where applicable, how potential secondary effects (e.g. second site T-DNA insertions, mosiacism, off-target gene editing) were examined.

Flow Cytometry

Plots

Confirm that:

☒ The axis labels state the marker and fluorochrome used (e.g. CD4-FITC).

☒ The axis scales are clearly visible. Include numbers along axes only for bottom left plot of group (a 'group' is an analysis of identical markers).

☒ All plots are contour plots with outliers or pseudocolor plots.

☒ A numerical value for number of cells or percentage (with statistics) is provided.

Methodology

Sample preparation

For immunostaining of cells expressing GPC proteins, we added monoclonal antibodies in binding buffer (1% bovine serum albumin [w/v] serum in PBS) for 60 min at 4 C. Cells were washed with binding buffer. After three with binding buffer, cells were incubated with the secondary antibody for 60 min at 4 C. Following incubation, we washed cells twice with binding buffer, twice with chilled PBS, fixed them with 2% (v/v) formalin, and detected cell surface expression or antibody binding by flow cytometry (PE positive cells).

Instrument

iQue3 Screener PLUS (Intellicyt).

Software

IntelliCyt ForeCyt Standard Edition version 8.1.7524 (Sartorius).

Cell population abundance

Flow cytometry was used for analysis but not for cell sorting.

Gating strategy

Gated for live cells with FSC-H and SSC-H. Gated for single cells with FSC-H and FSC-A. Then gated for fluorophore (PE).

☒ Tick this box to confirm that a figure exemplifying the gating strategy is provided in the Supplementary Information.
