# Supplementary figures and images for: Molecular organization of the New World arenavirus spike glycoprotein complex
Source: Nat Microbiol. 2025 Aug 8;10(9):2207–20. doi: 10.1038/s41564-025-02085-6 (PMC12408356; doi:10.1038/s41564-025-02085-6)

Extended Data Figure 1a

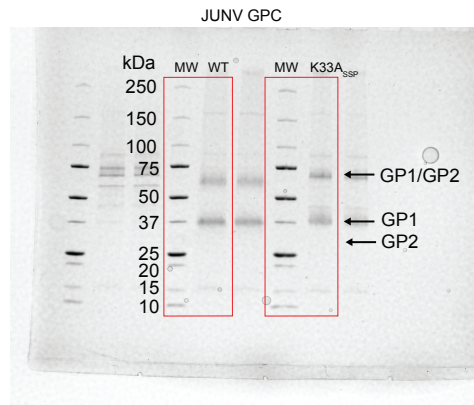

Extended Data Figure 1d

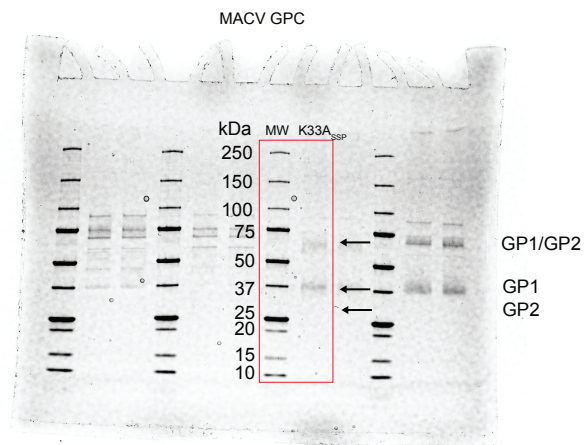

Supplement: Supplementary file 9 — Unprocessed gels. [file 41564_2025_2085_MOESM9_ESM.pdf]
